# Supplementary material for: Youth Culturally adapted Manual Assisted Problem Solving Training (YCMAP) in Pakistani adolescent with a history of self-harm: protocol for multicentre clinical and cost-effectiveness randomised controlled trial
Source: BMJ Open. 2022 May 12;12(5):e056301. doi: 10.1136/bmjopen-2021-056301 (PMC9109112; doi:10.1136/bmjopen-2021-056301)
Supplement: Supplementary data [file bmjopen-2021-056301supp001.pdf]

COVID-19 continuity plan

In March 2020, due to the first wave of global pandemic-COVID-19 there was a need to make amendments to study procedures in response to the lock down and social distancing in Pakistan. The trial during this period was in the internal pilot phase and in order to ensure smooth running of the project certain changes were made to continue with collection of data, follow ups and delivery of the intervention.

Follows ups (3rd, 6th, 9th and 12th month after randomization):

Initial ethical approval was obtained for face to face assessment and intervention. However, after COVID-19, we submitted ethics amendment for data collection and delivery of intervention digitally (i.e. through telephone call, zoom, skype or other remote means) so that the study team could continue their work and meet targets on time with working from home condition. The consent form that was previously completed on paper further consents will be obtained remotely and the assessment measures to be completed remotely as well. Recordings of consent will be stored in password protected systems.

YCMAP Intervention:

YCMAP intervention comprises of 8-10 sessions delivered in a face to face setting, usually in research offices, community clinics and homes (as per participant choice). As of 20th march 2020, due to lock down across Pakistan it was difficult to deliver face to face sessions. To comply with preventive measures such as social distancing and lock down guidelines, the YCMAP intervention will be delivered remotely, via a phone call, WhatsApp call, zoom or skype rather than in person face to face sessions. Contingency plan will be implemented to manage confidentiality and safe guarding risks.

Qualitative Interviews:

Qualitative interviews will be completed face to face or through telephone. Due to the pandemic situation COVID-19, topic guides will be revised in order to explore the experience of change in mode of delivery of intervention as well as impact of Covid 19 after discussing it with supervisors (EC, NC).

Supervision**:**

Arrangements for supervision of the therapists will continue as per protocol. The supervisions will continue to be carried out remotely via video link.

Trainings:

Arrangements for training of YCMAP team members will continue and carried out remotely via video link
