# Supplementary material for: Youth Culturally adapted Manual Assisted Problem Solving Training (YCMAP) in Pakistani adolescent with a history of self-harm: protocol for multicentre clinical and cost-effectiveness randomised controlled trial
Source: BMJ Open. 2022 May 12;12(5):e056301. doi: 10.1136/bmjopen-2021-056301 (PMC9109112; doi:10.1136/bmjopen-2021-056301)
Supplement: Author's manuscript [file bmjopen-2021-056301.draft_revisions.pdf]

BMJ Open

BMJ Open is committed to open peer review. As part of this commitment we make the peer review history of every article we publish publicly available.

When an article is published we post the peer reviewers' comments and the authors' responses online. We also post the versions of the paper that were used during peer review. These are the versions that the peer review comments apply to.

The versions of the paper that follow are the versions that were submitted during the peer review process. They are not the versions of record or the final published versions. They should not be cited or distributed as the published version of this manuscript.

BMJ Open is an open access journal and the full, final, typeset and author-corrected version of record of the manuscript is available on our site with no access controls, subscription charges or pay-per-view fees (http://bmjopen.bmj.com).

If you have any questions on BMJ Open's open peer review process please email info.bmjopen@bmj.com

BMJ Open

A Youth Culturally adapted Manual Assisted Problem Solving Training (YCMAP) in Pakistani adolescent with a history of self harm: Protocol for multi-centre clinical and cost effectiveness randomised controlled trial.

Journal:	*BMJ Open*
Manuscript ID	bmjopen-2021-056301
Article Type:	Protocol
Date Submitted by the Author:	16-Aug-2021

Complete List of Authors:	Husain, Nusrat; The University of Manchester School of Medical Sciences, Division of Psychology and Mental Health; Manchester Global Foundation<br>Tofique, Sehrish; Pakistan Institute of Living and Learning, Division of Neuro-Cognitive Disorder, Older Adults Mental Health<br>Chaudhry, Imran B.; Greater Manchester West Mental Hlth NHS Fdn Trust; Dr Ziauddin Hospital, Psychiatry<br>Kiran, Tayyeba; Pakistan Institute of Living and Learning , Division of Mood Disorder<br>Taylor, Peter; University of Manchester School of Psychological Sciences, Psychology & Mental Health<br>Williams, Christopher; University of Glasgow, Psychiatry<br>Memon, Rakhshi; University College London, Science and Technology Studies<br>Aggarwal, Shilpa; SRCC Children's Hospital (Narayana Health),, Psychiatry<br>Alvi, Mohsin; Pakistan Institute of Living and Learning, Health Economics<br>Ananiadou, S; University of Manchester, School of Computer Science<br>Ansari, Moin ; Sir Cowasjee Jehangir Institute Of Psychiatry<br>Aseem, Saadia; The University of Manchester, Division of Psychology and Mental Health<br>Beck, Andrew; Bradford Teaching Hospitals NHS Foundation Trust<br>Alam, Sumira; Services Institute of Medical Sciences, Psychiatry<br>Colucci, Erminia ; Middlesex University,<br>Davidson, Kate; University of Glasgow,<br>Edwards, Sarah; University College London, Research Ethics and Governance<br>Emsley, Richard; King's College London, Department of Biostatistics and Health Informatics, Institute of Psychiatry, Psychology and Neuroscience<br>Green, Jonathan; University of Manchester and Manchester Academic Health Sciences Centre, Division of Neuroscience &Experimental Psychology<br>Gumber, Anil; Sheffield Hallam University, Health Economics<br>Hawton, Keith; Centre for Suicide Research, Oxford University, Psychiatry<br>Jafri, Farhat; Karachi Medical and Dental College, Community Medicine<br>Khaliq, Ayesha; Pakistan Institute of Living and Learning , Division of Eating Feeding, Nutrition and Elimination Disorders<br>Mason, Thomas; University of Manchester, Division of Population Health,

	Health Services Research & Primary Care, Manchester Institute for Collaborative Research on Ageing<br>Mcreath, Ann; Lifestyle & Wellness<br>Minhas, Ayesha; The Tree House<br>Naeem, Farooq; University of Toronto, Psychiatry<br>Naqvi, Haider; Dow University of Health Sciences, Psychiatry<br>Noureen, Amna; Pakistan Institute of Living and Learning, Division of Child and Adolescent Mental Health<br>Panagioti, Maria; University of Manchester, Division of Population Health, Health Services Research & Primary Care<br>Patel, Anita; Queen Mary University of London,<br>Poppleton, Aaron; University of Manchester, Center for primary care and health services research,<br>Shiri, Tinevimbo; Liverpool School of Tropical Medicine, Health Economics<br>Simic, Mima; South London and Maudsley NHS Foundation Trust<br>Sultan, Sarwat; Bahauddin Zakariya University, Department of Applied Psychology<br>Nizami, Asad; Institute of Psychiatry, Psychiatry<br>Zadeh, Zainab; Pakistan Institute of Living and Learning, Child and Adolescent Mental health<br>Zafar, Shehla; Iqra University<br>Chaudhry, Nasim; Pakistan Institute of Living and Learning, Research and Development

Keywords:	Suicide & self-harm < PSYCHIATRY, Child & adolescent psychiatry < PSYCHIATRY, MENTAL HEALTH

SCHOLARONE™
Manuscripts

A Youth Culturally adapted Manual Assisted Problem Solving Training (YCMAP) in Pakistani adolescent with a history of self harm: Protocol for multi-centre clinical and cost effectiveness randomised controlled trial.

- Corresponding author (Tayyeba kiran,75600,tayyebakiran@gmail.com, tayyaba.kiran@pill.org.pk)
- Co-authors (full name, dept, institution, city and country)

Prof. Nusrat husain[1] , Division of Psychology and Mental Health, University of Manchester, Manchester, UK
Manchester Global foundation, Manchester, UK
Sehrish Tofique[2] , Division of Neuro-cognitive Disorders, Older Adults Mental Health, Pakistan Institute of Living and learing, Karachi, Pakistan
Prof. Imran Bashir Chaudhry[3] ,Professor of Psychiatry, University of Manchester, Manchester, UK.
Dept of Psychiatry, Ziauddin Hospital, Karachi, Pakistan
Tayyeba Kiran[2] , Division of mood disorder, Pakistan Institute of Living and Learing, Karachi, Pakistan
Dr.Peter Taylor[1] , Division of Psychology and Mental Health, University of Manchester, Manchester, UK
Prof. Christopher Williams[4] ,Professor of Psychosocial Psychiatry , University of Glasgow, Scotland, UK
Rakhshi Memon[5] , Department of Science and Technology Studies, University College London,UK
Dr Shilpa Aggarwal[6], Child and Adolescent Psychiatrist, SRCC Children's Hospital (Narayana Health), Mumbai , India
Mohsin Hassan Alvi[2] , Department of Health Economics,Pakistan Institute of Living and Learing, Karachi, Pakistan
Prof. Sophia Ananiadou[1], School of Computer Science, University of Manchester, Manchester, UK
Prof. Moin Ansari[7], Dept of Psychiatry LUMHS, Sir Cowasjee Jehangir Institute of Psychiatry, Karachi, Pakistan
Dr Saadia Aseem[1] , Division of Psychology and Mental Health, University of Manchester, Manchester, UK
Dr Andrew Beck[8], Clinical Psychologist, Head of Clinical Health Psychology, Bradford Teaching Hospitals, NHS Foundation Trust
Dr Sumira Qambar Bokhari[9], Department of Psychiatry,Services Institute Of Medical Sciences, Lahore, Pakistan
Dr Erminia Colucci[10], Depart ent of Psychology, Middlesex University,London, UK
Prof. Kate Davidson[4], Institute of Health and Wellbeing, University of Glasgow, Scotland, UK
Dr Sarah Jane Louise Edwards[5], Research Ethics and Governance, University College London, UK
Prof.Richard Emsley[11], Biostatistics & Health Informatics, Institute of Psychiatry, Psychology and Neuroscience,Kings College London, UK
Prof. Jonathan Green[1],Division of Neuroscience &Experimental Psychology,University of Manchester, Manchester, UK
Dr. Anil gumber[12], Principal Health Economist, Sheffield Hallam University, UK
Prof. Keith Hawton[8], Psychiatry, Oxford Health NHS Foundation Trust, University of Oxford, UK
Prof. Farhat Jafri[13], Community Medicine, Karachi Medical and Dental College, Karachi, Pakistan
Ayesha Khaliq[2] , Division of Eating Feeding, Nutrition and Elimination Disorders , Pakistan Institute of Living and Learning, Lahore, Pakistan
Dr. Thomas Mason[1], Division of Population Health, Health Services Research & Primary Care, Manchester Institute for Collaborative Research on Ageing, Manchester, UK
Ann C McCreath[14], Mental Health Nurse, Director at Lifestyle & Wellness, UK
Prof. Ayesha Minhas[15], Psychiatry, The Tree House, Rawalpindi, Pakistan
Prof.Farooq Naeem[16], Psychiatry, University of Toronto, Toronto, Canada
Prof. Haider Ali Naqvi[17], Psychistry, Dow University of Health Sciences, Karachi, Pakistan
Amna Noureen[2], Division of Child and Adolescents Mental health, Pakistan Institute of Living and Learning, Rawalpindi, Pakistan

For peer review only - http://bmjopen.bmj.com/site/about/guidelines.xhtml

Dr. Maria Panagioti[1],Division of Population Health, Health Services Research & Primary Care, Institute for Health Policy and Organisation /Alliance Manchester Business School, Manchester, UK
Prof.Anita Patel[18], Health Economics, Queen Mary, University of London, UK
Dr. Aaron Poppleton[1],NIHR Academic Clinical Fellow in General Practice, Center for Primary Care and Health Services Research,University of Manchester
Tinevimbo Shiri[19], Health Economics, Liverpool School of Tropical Medicine, England, UK
Dr. Mima Simic[20], Child and Adolescent Psychiatrist, The Maudsley Hospital London,NHS foundation England, United Kingdom
Dr. Sarwat Sultan[21], Division of Psychology, Bahauddin Zakaria University, Multan, Pakistan
Prof. Asad Tamizuddin[22], Psychiatry, Institute of Psychiatry, Rawalpindi, Pakistan
Prof. Zainab Fotowwat Zadeh[2] , Division of Child and Adolescent health, Pakistan Institute of Living and Learning, Karachi, Pakistan
Prof. Shehla Naeem Zafar[23], Community and Public Health Nursing, Iqra University, Karachi, Pakistan
Prof. Nasim Chaudhry[2] , Research and Development, Pakistan Institute of Living and Learning, Karachi, Pakistan

- Word count (excluding title page, abstract, references, figures, tables-4165/4000)
- Start & End Date of Study: March 2019 – Feburary 2023

For peer review only

For peer review only - http://bmjopen.bmj.com/site/about/guidelines.xhtml

ABSTRACT

Introduction: Suicide is a global health concern. Socio-cultural factors have an impact on self-harm and suicide rates. In Pakistan both self-harm and suicide are considered as criminal offense's and are condemned on both religious and social grounds. The proposed intervention "Youth Culturally Adapted Manual Assisted Problem Solving Training (YCMAP)" is based on principles of problem-solving and cognitive behavioural therapy (CBT). YCMAP is a brief, culturally relevant, scalable intervention that can be implemented in routine clinical practice if found to be effective.

Method and Analysis: A multi-centre rater blind randomized control trial (RCT) to evaluate the clinical and cost effectiveness of YCMAP including a sample of 652 participants, aged 12-18 years, presenting to general physicians/clinicians, emergency room after self harm or self referrals. We will test the effectiveness of 8 to 10 individual sessions of YCMAP delivered over three months compared to Treatment as Usual (TAU). Primary outcome measure is repetition of self-harm at 12 months. The seconday outcomes include reduction in suicidal ideation, hopelessness and disress and improvement in health related quality of life. Assessments will be completed at baseline, 3, 6, 9 and 12 month post randomization. The nested qualitative component will explore perceptions about management of self-harm and suicide prevention amongst adolescents and investigate participants' experiences with YCMAP. The study will be guided by the Theory of Change approach to ensure that the whole trial is centred around needs of the end beneficiaries as key stakeholders in the process.

Ethics and Dissemination: Ethics approval has been obtained from the Ethics Committee of University of Manchester, the National Bioethics Committee in Pakistan. The findings of this study will be disseminated through community workshops, social media, conference presentations and peer-reviewed journals.

Trial Registration Number: NCT04131179

For peer review only - http://bmjopen.bmj.com/site/about/guidelines.xhtml

STRENGTHS AND LIMITATIONS OF THIS STUDY

1. This is the first multi centre RCT to evaluate clinical and cost effectiveness of a culturally relevant psychological intervention for young people presenting with self-harm in a Lower and Middle Income Country (LMIC).
2. This trial fulfills an important clinical need of LMICs, specifically in Pakistan.
3. The trial will provide evidence based outcome data to inform and support policy makers in formulating policies for prevention of self-harm and suicide.
4. Stigma, fear of persecution by authorities, peer pressure and educational commitments may act as a barriers to young people participating in the study.
5. The Theory of Change approach will help mitigate some of the risks around stigma and refusal to participate by involving parents and young people from the inception of the trial.

INTRODUCTION

Suicide is one of the major public health concerns globally with 800,000 suicides each year across the globe (1). More than 75% of suicides occur in Low and Middle Income Countries (LMICs). Overall, suicide is the 2nd leading cause of death in individuals between 15-29 years old (1). A recent review reported suicide rates in South Asia to be higher than the global average(2). However, such figures are likely to be an underestimate due to a lack of accurate data on suicide in Pakistan (3) and in many other LMICs (4).

In Pakistan, a conservative Islamic state, policies are changing but suicide and self-harm (defined below) remain criminal acts and are condemned both socially and religiously as a moral wrong. Suicide and self-harm are often considered taboo subjects across the country, thus contributing to a lack of evidence and under-reporting (2). There is, however, accumulating evidence that both self-harm and suicide rates have been increasing in Pakistan but there continues to be notable gaps in evidence(2). In a systematic review of mental health studies of adolescents in India, it was found that self-harm is particularly problematic in young people, with three months prevalence ranging from 3.9% to 25.4% in community based studies (5, 6). Self-harm is one of the strongest predictors of death by suicide (7). Self-harm also carries a substantial economic impact and has been associated with large treatment costs in Pakistan (8). Adolescents with a history of self-harm are at a higher risk of repeating this later in life (9)thus there is an important need to develop effective interventions in order to avoid long-term burden.

In adolescents, self-harm is the result of a highly complex interplay between genetic, biological, psychological, social, and cultural factors (9). Common set of risk factors and psycho-social mechanisms for self-harm within LMICs include being female, experiencing interpersonal conflict, suffering from abuse (including domestic/family/gender based violence), hopelessness, and being diagnosed with psychiatric disorder (2). Other psychological influences include feelings

For peer review only - http://bmjopen.bmj.com/site/about/guidelines.xhtml

For peer review only

of entrapment, a lack of belonging or connection (10). Research in Pakistan is imperative to explore the effectiveness of interventions for potential risk factors to reduce rates of self-harm and suicide (11). The World Health Organization (WHO) Report "preventing Suicide: A Global imperative" recommends "two-fold" public health approach for suicide prevention i.e. to identify the issues, and provide treatment for high-risk individuals. Considering the benefits of adult individual Cognitive Behaviour Therapy (CBT) -based psychotherapy, there is a need to develop this further for adolescents (12). Psychological therapies that work in western culture cannot always be implemented in a different culture and adaptation is considered to be essential (13, 14).

Since interpersonal conflicts with family members are a commonly reported precipitant of self-harm episodes in Pakistan (2, 11). It has been reported that problem-solving therapy "Culturally adapted Manual Assisted brief Psychological intervention (CMAP)" may be a useful intervention for prevention of self-harm in Pakistan (11). This psychological intervention, using variants of CBT that are age appropriate for the local culture and customs in Pakistan, may prove to be beneficial in reducing self-harm in adolescents (15). Before this intervention can be implemented at a larger scale, there is a need for establishing its efficacy and cost-effectiveness via the proposed trial. We cannot assume that an intervention developed for adults would be suitable for children and adolescents and since self-harm remains a major problem in young people, this planned trial fills an important gap and clinical need.

METHODS AND ANALYSIS

Objectives

This study includes both quantitative and qualitative aspects.

The objectives of the quantitative component are:

1. To determine the clinical effectiveness of YCMAP over 12 months based on the following outcomes:
    a. Primary outcome:
        i. To measure the effectiveness of YCMAP in comparison to treatment as usual (TAU) in terms of repetition of self-harm 12-month post randomization. This will be assessed using adapted Suicide Attempt Self-Injury Interview SASII (16).
    b. Secondary outcomes:
        i. Suicidal ideation will be assessed using the "Beck Scale for Suicidal ideation (BSI)"(17).

For peer review only - http://bmjopen.bmj.com/site/about/guidelines.xhtml

ii. Feelings of hopelessness will be assessed using the "Beck Hopelessness Scale (BHS)"(18).
iii. Level of distress will be assessed using a 10-item scale the "Kessler Psychological Distress Scale (K10)"(19).
iv. EQ-5D- Y will be used to assess "health related quality of Life" (20).
v. Client Satisfaction Questionnaire-"CSQ-8" will be used to assess participants' satisfaction with services (21).

Translated versions of all these scales have been developed and used in previous trials CMAP1 (15) and CMAP 2 (11) by following established protocols for translating such measures (22).The scales which were not available in Urdu were translated for an MPhil project (Brief psychological intervention for adolescents who self harm, IPP/BU/ER/103/1377) and reviewed in initial Patient and Public Involvement and Engagement meetings.

Assessments will be carried out at baseline, completion of the intervention (3 months), 6, 9 and 12 months after randomisation. See figure 1 below

2. To determine the cost effectiveness of the YCMAP intervention over 12 months.

The objectives of the qualitative component are:

1. To explore the experience of participants with YCMAP and their perception about benefits and negative or adverse consequence of it.
2. To explore in detail participants' reasons for continuing (completer) or not continuing (drop out) with the study.
3. To explore clinicians and other stakeholder views (such as school teachers) on the YCMAP and its impact.
4. To explore therapists' perspective on the delivery of the YCMAP.

The expected outputs of the study are:
a) A manual-assisted evidence based intervention - the YCMAP, ready for integration into the health care services.

b) Standardized training and supervision package - a resource pack and training program on 'how to do it' for use across health services.

c) Information Tool-kit for the families and the wider community. This will be service-user defined and will be used to increase awareness about mental health in general and self-harm and suicide in young people in particular.

For peer review only - http://bmjopen.bmj.com/site/about/guidelines.xhtml

Inclusion Criteria

Adolescets meeting the study inclusion criteria will be invited to take part in the study by their primary care clinician, ward or emergency room (ER) clinician who is making the initial assessment. In the context of this study, self-harm is defined as: *"an act with non-fatal outcome, in which an individual deliberately initiates a non-habitual behaviour that, without interventions from others, will cause self-harm, or deliberately ingests a substance in excess of the prescribed or generally recognised therapeutic dosage, and which is aimed at realizing changes which the subject desired via the actual or expected physical consequences"(23).*

- Age: 12-18 years.
- History of recent self-harm which is defined as " self-harm occurring within the last 3 months (from the initial identification of a potential participant)". This period is considered as high risk for repetition in young people (9).
- Resident in the trial catchment area.
- All participants known to clinical or health services. This will be necessary to ensure all participants have access to TAU.

Exclusion Criteria

- Patients with a severe mental illness, such as psychotic disorder.
- Patients with conditions limiting engagement with assessment/intervention.
- Temporary resident unlikely to be available for follow up.

Design

A multi-centre rater blind RCT with randomisation by individual participants in order to compare the YCMAP in addition to TAU with TAU alone.

Study Site and Population

Study sites are all participating primary care clinics,emergency departments and medical wards of general hospitals in five major cities of Pakistan, Karachi, Hyderabad, Lahore, Multan and Rawalpindi.

Sample Size

The sample size is based on the primary outcome, repetition of self-harm in a 12-month period (yes/no). The TAU arm of the study has an expected self-harm rate of 20% (5). A clinically important effect would be a reduction to 7.5% in the intervention group. Under these presumptions,

For peer review only - http://bmjopen.bmj.com/site/about/guidelines.xhtml

and assuming a 5% significance level and 90% power, a study with no clustering would require 158 patients per arm. The study has a partially nested design due to therapist clustering in the YCMAP arm. Our sample size calculation has taken account of this by adjusting the sample size upwards, conservatively assuming clustering in both arms. Based on previous analysis of therapist trials, we believe that the intraclass correlation coefficient (ICC) is likely to have a value between 0.01 and 0.05 for this type of outcome measure. Assuming an ICC of 0.05, and a cluster size of 16 patients per therapist, a design effect of 1.75 is calculated. This increases the numbers required to 277 per arm. Furthermore,based on our previous work there is expected to be a 15% loss to follow-up, and so the final numbers recruited will be 326 per arm, with a total sample of 652 participants.

For peer review only

Randomisation

The researcher will complete a checklist, confirming eligibility, obtain written (or verbal audio-recorded consent from the subject due to current pandemic COVID-19 SOPs), and complete the baseline measures. The researcher will contact the randomisation centre, who will re-check eligibility, record baseline measures and assign a participant's trial number. Treatment assignment will then be determined using stochastic minimisation controlling for gender, age and type of self-harm behaviour. The independent statistician would inform the project manager of the random assignments, but this information would not be shared with research staff, so that they remain unaware of whether a particular participant receives YCMAP or not. Participants assigned to the YCMAP would then be contacted by a therapist, who will arrange an initial meeting within 2-4 weeks after baseline to start the intervention. Participants in the TAU group would be informed of their allocation to this group after randomisation had taken place.

Recruitment and Baseline Assessment

Potentially eligible individuals will be identified by clinical staff at the participating recruitment sites. An age appropriate version of the Participant Information Sheet (PIS) will be provided alongside the standard PIS, so both the parent/guardian and child can access this information. For individuals who cannot read, information about the study will be summarised by the clinician for them.

Intervention

YCMAP is a "Youth culturally adapted manual assisted psychological intervention" based on CBT principles. It comprises of 8-10 sessions delivered over three months.The first eight sessions are offered weekly and further sessions fortnightly on a one to one basis and each session lasts for about 60 minutes. The YCMAP has been culturally adapted with permission from "CMAP" (15), "Life after self-harm" (24) and "Cutting down: A CBT workbook for treating young people who

For peer review only - http://bmjopen.bmj.com/site/about/guidelines.xhtml

self-harm”(25). The intervention includes psycho-education and a comprehensive cognitive behavioural assessment of the self-harm attempt using virtual stories of four young people. The therapy focuses on current problems that contributed to the self-harm episode. Therapists and adolescent clients choose from a list of techniques those which are most relevant to the client’s problems. Therapy is therefore adapted to fit with the clients problems and primarily utilises problem solving, CBT, and dialectical therapy strategies to bring about change.

To help determine the most appropriate coping strategy a coping tree is designed. Training in assertiveness and anger management are offered to help the young person to develop resilience to cope with stress. To the existing YCMAP we will also aim to review the look and feel of the resource by including licenced content from My Big Life - a course developed by our collaborative partner Five Areas Limited. This accessible, story-based approach uses illustrations of young people facing different scenarios at home and at school to illustrate key CBT-based concepts: Understanding your feelings -How to get a Big Life (behavioural activation) - Thinking in a Big Life way (identifying and changing thoughts that upset and affect how you feel) - Relaxation approaches - Building inner confidence - Practice scenarios - Trainer notes and linked worksheets/prompt cards and posters. Asian versions of the course already exist with amended artwork. As part of the development programme selected content will be added to the course and trainer notes will be modified (based on feedback) as needed.

All research staff will be required to have at least an undergraduate level degree in a relevant area (e.g. psychology) and will be trained in “Good Clinical Practice (GCP) ” in research, consent process, and use of the assessment measures. Regular supervision meetings will be arranged for research staff and therapists for case discussions, identifying and managing distress in participants. A study protocol will be in place prior to the start of the trial. This will include details about how to manage difficult situations arising in research, safety and lone working arrangements.

Treatment as Usual (TAU)

Local psychiatric, medical and primary care services provide standard routine care according to their clinical judgment and available resources. We will administer Client Services Receipt Interview (CSRI) (26) to obtain details regarding treatment received by each study participant.These participants will receive an initial assessment along with TAU as ascertained by their treating doctor at the hospital or their GP. The current practice is that self-harm patients are not routinely referred to mental health services. Research staff will record the nature and intensity of the routine care delivered for each participant.

For peer review only - http://bmjopen.bmj.com/site/about/guidelines.xhtml

Statistical Analysis

Statistical analysis will be based on intention-to-treat principles, subject to the availability of data. During the course of the trial, periodic random quality checks of data will be carried out by the trial statistician blind to treatment allocation. Once data entry has been completed, preliminary data analysis will be carried out blind to treatment allocation, prior to un-blinding. The results of the trial will follow the standard CONSORT recommendations. Baseline and follow-up data will be summarised using the appropriate descriptive statistics and graphical summaries. Treatment effects will be presented with 95% confidence intervals. We will investigate baseline factors that predict nonresponse using a logistic random effects model as non-response may be clustered by therapy group. The statistical analysis of the primary outcome measure (repetition of the self-harm episode within 12-months) will be based on a logistic random effects model with randomised treatment, age, gender and type of self-harm as fixed effects and a random effect of therapist in the treatment arm, with individuals in the control arm being treated as clusters of size 1. The continuous secondary outcome measures will calculate treatment effects using a linear mixed model, with a random effect of therapist and the set of baseline covariates as above including baseline values of the outcome where available. A single model will be fitted across all time-points, with a fixed effect for time, and interactions between time and treatment group. There will be no adjustment to secondary outcomes CIs for multiple testing. Binary secondary outcomes will take a similar approach to continuous outcomes, but will use logistic random effects models.

Economic Evaluation

The economic evaluation will take a societal perspective that incorporates the costs of both formal and informal health care and other relevant economic impacts such as on education. It will include financial impacts on providers and out of pocket payments by participants and their families. The use and payment for services will be collected using the CSRI (26) with patients and their families at baseline and at each follow up assessment. The use of the YCMAP intervention will be recorded separately by the trial team. Appropriate unit costs will be collected and attached to individual-level resource use quanitities to estimate total care costs for each individual. Cost estimates for the YCMAP intervention will include a) the salary of the therapists and b) indirect costs like session preparation, supervision, on-costs and capital overheads. Comparisons of total costs between the YCMAP and TAU arms will be based on non-parametric bootstrapped regressions (with co-variates for baseline costs, outcome measures and other key baseline factors) to account for the likely non-normal distribution in costs. An inital analysis will examine whether any additional costs associated with the YCMAP intervention are offset by savings elsewhere.

More formal assessments of cost-effectiveness will link between-arm differences in average costs with between-arm differences in a) the primary outcome measure, repetition of self-harm (SASII) and b) quality adjusted life year (QALY) gains. QALYs will be estimated by applying relevant

For peer review only - http://bmjopen.bmj.com/site/about/guidelines.xhtml

utility weights to health states measured through EQ-5D-Y (20, 27). Costs and outcomes will be linked in the form of incremental cost-effectiveness ratios (ICERs) where relevant, and cost-effectiveness acceptability curves (CEACs) based on the net benefit approach. As per the clinical effectiveness analyses, we will use an intention-to-treat approach for all economic analyses.

Qualitative Data Analysis

A purposefully selected subset (stratified by age, gender and self-harm severity) of participants in the treatment group will be invited to complete a qualitative one-to-one digitally recorded interview (face to face or via phonecall) lasting 1-1.5 hours. This will focus on their experience with the YCMAP intervention, the barriers and facilitators to engagement and perceived positive or negative experience with the intervention. A sample of 12-20 participants is likely to be sufficient to ensure data saturation (28) (29). Focus groups ($k = 2$ with 8-10 participants each) with therapists and key stakeholders will also be conducted to enable a further process analysis concerning the wider implementation of YCMAP into the Pakistani health systems. During both the individual and group interviews, broader suggestions for management and prevention of self-harm among young Pakistanis will also be collected to inform future service and research development.

We will explore perspectives of therapists and their supervisors about training and delivery of the model of care. We will also interview other key stakeholders to establish facilitators and barriers to implementation of the intervention in Pakistan.Targeted interviews will be done with a sample of participants who completed the intervention and a sample of participants who 'dropped out' before completion. Up to 30 interviews are likely to be needed with patient participants, to achieve category saturation. New text mining tools will be used to link heterogeneous data, to ensure that knowledge scattered with in lengthly textual data reaches clinicians, patients and policy makers to support decision making using different sources of information (30). Qualitative data will be analysed using Thematic Analysis (31) adopting a critical realist perspective (32).This will enable the research team to draw conclusions on the intervention whilst accounting for contextual and cultural factors.

Topic guides will be developed through discussion amongst trained qualitative researchers as well as with reference to relevant literature. Semi- structured interviews will be done to explore views on the “effectiveness and sustainability” of the YCMAP intervention in the management of people presenting with self-harm. Written or verbal consent at the time of interview (face to face or telephone) or prior to the interview (considering current pandemic) will be obtained from study participants and stake holders.

For peer review only - http://bmjopen.bmj.com/site/about/guidelines.xhtml

Internal pilot study

We have included an internal pilot phase (initial 12 months) with clear stop/go criteria across the study centres. This is included to ensure achievement of recruitment targets is viable across all sites included in this full trial. Progression to the full trial will be dependent on meeting the key objectives and stop/go criteria outlined below. The pilot phase will examine the processes for recruitment and logistical practices to support the effective execution of the full RCT. The internal pilot phase will be conducted in line with the National Institute of Health Research (NIHR) guidelines (33). The key objectives of the pilot are as follows; i) Recruitment of sufficient number of General Practitioners/Hospital Departments to support the trial. ii) Identify the proportion of eligible participants recruited across the study centres, and examine the feasibility of achieving recruitment targets to a full multicenter RCT. The pilot will be judged successful if sufficient GPs and hospitals are recruited to enroll 200 participants in the first year of recruitment i.e 4-12 months.We will monitor recruitment and identify the reasons for any shortfall on a monthly basis. The predetermined Stop-Go criteria is as follows:

i. Go if successfully recruit 180 (target minus 10%) or more participants during pilot study phase

ii. Rescue plan to be implemented if less than 180 but greater than 120 participants recruited in pilot phase.

iii. Stop if less than 120 participants recruited into the trial.

Theory of Change (ToC)

The study team uses ToC as the standard framework for all research projects. The overall study will be underpinned by the ToC causal model of planning, monitoring, evaluation and impact assessment to ensure that marginalised voices are included in developing the vision, identifying barriers and challenges from the lens of the end beneficiaries and short, medium and long term outcomes are developed and delivered so real change happens from the perspective of the target group. A ToC workshop with key stakeholders has already taken place to ensure all stakeholders have a buy in and ownership of the process. See Figure 2

The ToC causal diagram as an output is evidence of stakeholder engagement and ownership. Also,ToC will enable us to show how trial results are adopted in wider practice and on what basis,what barriers and challenges were faced during the trial and what assumptions were made in defining the goal statements? Informed by the ToC workshop, a Young People's Advisory Group will be set up to provide insight into the lived experiences of today's youth in Pakistan.

For peer review only - http://bmjopen.bmj.com/site/about/guidelines.xhtml

Patient and Public Involvement and Engagement (PPIE) Group

This group will help to ensure that the research agenda for self-harm and suicide prevention studies is informed by and aligned with service users and carer priorities.

Trial Steering Committee (TSC)

We will convene an independently chaired TSC to approve and provide oversight of the trial throughout its various stages. The TSC will include the chair, user representative, three PIs, an independent statistician and a representative from the local health department. This will be independent of the trial management team. The (TSC) will convene annually, but twice in the first year, with feedback from the Chair as and when needed.

Data Monitoring and Ethics Committee (DMEC)

The DMEC will oversee the data and advise the TSC on any ethical or safety concerns.

Ethics

Ethics approval for the trial has been obtained from the Research Ethics Committee of University of Manchester (Ref: 2019-5024-10755) and the National Bioethics Committee in Pakistan (Ref: No.4-87/NBC-419/19/1213).

Dissemination

The study findings will be disseminated using social media, at national and international conferences in partnership with the "service user", "carer" and "community organizations". Also, study results will be submitted to peer and non peer-reviewed journals for publication.

Discussion

There has been a recent significant increase in number of trials related to self-harm and suicide prevention which reflects the need and international concern about self-harm/suicide prevention. However, in Pakistan evidence based research is limited with no specific intervention for adolescents with self harm history. The evidence suggests that CMAP intervention for adults is effective in Pakistan (15,34). The components of CMAP intervention may also be beneficial for adolescents presenting after self-harm, therefore there is a strong need to determine the effectiveness of culturally adapted manual assisted problem solving intervention for adolescents in Pakistan. Findings will also contribute to evidence based treatments for self-harm and will meet

For peer review only - http://bmjopen.bmj.com/site/about/guidelines.xhtml

important clinical needs of Pakistan and will help guide policy makers in developing suicide prevention policies.

Authors Contributions

Prof. NH, NC, TK involved in conceptualisation, designing and planning of the project (including protocol development). All authors gave their valuable inputs in finalizing the study protocol.

Funding Statement

The Medical research Council /DFID/NIHR programme (MR/R022461/1) has funded this trial. The funder has no role in designing the study methodology, data analysis and interpretation, manuscript writing and in the decision to submit the protocol for publication.

Competing Interests

All authors have read and declared that we have no competing interests with the exception of NH, NC, IBC, TK and CW. NH, former Trustee of "Pakistan Institute of Living and Learning (PILL)", "Abaseen Foundation (UK)" and "Lancashire Mind (UK)". At "Manchester Global Foundation", he is the Chair of Board of Trustees. He is also a member of the executive committee for the Faculty of Academic Psychiatry, at the Royal College of Psychiatrists. NH is a NIHR Senior Investigator. NC is Associate Director of Global Mental Health and Cultural Psychiatry Research Group. IBC, former Trustee of "PILL" is Honorary Professor at the University of Manchester. NH, IBC and NC have received support for educational programs and/or travel support and/or speaker fees from pharmaceutical companies. CW is the author of a book aimed at suicide prevention, and has written a range of books and online CBT-based course resources that are available as both, free access and on a commercial basis. NH, NC, IBC and TK's time is partially funded by the Global Challenges Research Fund "South Asia Harm Reduction Movement-SAHAR M" (MR/P028144/1).

Acknowledgement

We express our gratitude to all team members, researchers, consultants and investigators for their support and advice in the development and setting up of the trial. We would like to thank community gate keepers, service uers and patient and public involvement group members for their kind support, input and feedback on the development of the funding application, spreading awareness among masses and in trial recruitments.

For peer review only - http://bmjopen.bmj.com/site/about/guidelines.xhtml

We would also like to thank Trial Steering Committee (TSC) members, Prof. David Kingdon (Chair), Dr. Mehmood Shaukat, Faraz Usman and Data Management and Ethics Committee (DMEC) members Prof. Unaiza Niaz (Chair), Dr. Qamar Saeed and Dr. Maryum Ilyas for overseeing the trial's progress and management.

Appendix – COVID-19 continuity plan

In March 2020, due to the first wave of global pandemic-COVID-19 there was a need to make amendments to study procedures in response to the lock down and social distancing in Pakistan. The trial during this period was in the internal pilot phase and in order to ensure smooth running of the project certain changes were made to continue with collection of data, follow ups and delivery of the intervention.

Follows ups (3rd, 6th, 9th and 12th month after randomization):

Initial ethical approval was obtained for face to face assessment and intervention. However, after COVID-19, we submitted ethics amendment for data collection and delivery of intervention digitally (i.e. through telephone call, zoom, skype or other remote means) so that the study team could continue their work and meet targets on time with working from home condition. The consent form that was previously completed on paper further consents will be obtained remotely and the assessment measures to be completed remotely as well.Recordings of consent will be stored in password protected systems.

YCMAP Intervention:

YCMAP intervention comprises of 8-10 sessions delivered in a face to face setting, usually in research offices, community clinics and homes (as per participant choice). As of 20th march 2020, due to lock down across Pakistan it was difficut to deliver face to face sessions.To comply with preventive measures such as social distancing and lock down guidelines, the YCMAP intervention will be delivered remotely, via a phone call, whatsapp call, zoom or skype rather than in person face to face sessions. Contingency plan will be implemented to manage confidentiality and safe guarding risks.

Qualitative Interviews:

Qualitative interviews will be completed face to face or through telephone. Due to the pandemic situation COVID-19, topic guides will be revised in order to explore the experience of change in mode of delivery of intervention as well as impact of Covid 19 after discussing it with supervisors (EC,NC).

For peer review only - http://bmjopen.bmj.com/site/about/guidelines.xhtml

Supervision:

Arrangements for supervision of the therapists will continue as per protocol. The supervisions will continue to be carried out remotely via video link.

Trainings:

Arrangements for training of YCMAP team members will continue and carried out remotely via video link

References:

1. WorldHealthOrganization. Preventing suicide: A global imperative: World Health Organization; 2014. https://www.who.int/publications/i/item/9789241564779
2. Shekhani SS, Perveen S, Akbar K, et al. Suicide and deliberate self-harm in Pakistan: a scoping review. BMC psychiatry. 2018;18(1):1-15. https://DOI 10.1186/s12888-017-1586-6
3. Farooq U, Majeed M, Bhatti JA, et al. Differences in reporting of violence and deliberate self harm related injuries to health and police authorities, Rawalpindi, Pakistan. 2010;5(2). https://doi.org/10.1371/journal.pone.0009373.
4. Jordans MJ, Kaufman A, Brenman NF, et al. Suicide in South Asia: a scoping review. 2014;14(1):358. https:// DOI 10.1186/s12888-014-0358-9
5. Aggarwal S, Berk M. Evolution of adolescent mental health in a rapidly changing socioeconomic environment: A review of mental health studies in adolescents in India over last 10 years. J Asian journal of psychiatry 2015;13:3-12. https://doi.org/10.1016/j.ajp.2014.11.007
6. Aggarwal S. Suicide in India. J British medical bulletin. 2015;114(1). https:// doi: 10.1093/bmb/ldv018
7. Ribeiro J, Franklin J, Fox KR, et al. Self-injurious thoughts and behaviors as risk factors for future suicide ideation, attempts, and death: a meta-analysis of longitudinal studies. 2016;46(2):225-36. https://doi:10.1017/S0033291715001804
8. Shahid M, Khan MM, Naqvi H, et al. Cost of treatment of deliberate self-harm: a study from Pakistan. 2008;29(4):213-5. https://doi.org/10.1027/0227-5910.29.4.213
9. Hawton K, Saunders KE, O'Connor RC. Self-harm and suicide in adolescents. The Lancet Neurology. 2012;379(9834):2373-82. https://doi.org/10.1016/S0140-6736(12)60322-5
10. Klonsky ED, May AM. The three-step theory (3ST): A new theory of suicide rooted in the “ideation-to-action” framework. International Journal of Cognitive Therapy. 2015;8(2):114-29. https://doi.org/10.1521/ijct.2015.8.2.114
11. Husain MO, Umer M, Taylor P, et al. Demographic and psychosocial characteristics of self-harm: The Pakistan perspective. Psychiatry research. 2019;279:201-6. https://doi.org/10.1016/j.psychres.2019.02.070
12. Witt KG, Hawton K, Hetrick S, et al. Interventions for self-harm in children and adolescents. 2020. https://DOI: 10.1002/14651858.CD013667.

For peer review only - http://bmjopen.bmj.com/site/about/guidelines.xhtml

13. Rathod S, Gega L, Degnan A, et al. The current status of culturally adapted mental health interventions: a practice-focused review of meta-analyses. 2018;14:165. https://www.ncbi.nlm.nih.gov/pmc/articles/PMC5757988/
14. Degnan A, Baker S, Edge D, et al. The nature and efficacy of culturally-adapted psychosocial interventions for schizophrenia: a systematic review and meta-analysis. 2018;48(5):714-27. https://DOI: 10.1017/S0033291717002264.
15. Husain N, Afsar S, Ara J, et al. Brief psychological intervention after self-harm: randomised controlled trial from Pakistan. The British Journal of Psychiatry. 2014;204(6):462-70. https://doi: 10.1192/bjp.bp.113.138370.
16. Linehan MM, Comtois KA, Brown MZ, et al. Suicide Attempt Self-Injury Interview (SASII): development, reliability, and validity of a scale to assess suicide attempts and intentional self-injury. Psychological assessment. 2006;18(3):303. https://doi.org/10.1037/1040-3590.18.3.303
17. Beck AT, Steer RA. Manual for the Beck scale for suicide ideation. San Antonio, TX: Psychological Corporation1991;63.
18. Beck AT, Steer RA, Pompili M. BHS, Beck hopelessness scale: manual: Psychological corporation San Antonio, TX; 1988.
19. Andrews G, Slade T. Interpreting scores on the Kessler psychological distress scale (K10). Australian and New Zealand journal of public health. 2001;25(6):494-7. https://doi.org/10.1111/j.1467-842X.2001.tb00310.x
20. Ravens-Sieberer U, Wille N, Badia X, et al. Feasibility, reliability, and validity of the EQ-5D-Y: results from a multinational study. Quality of life research. 2010;19(6):887-97. https://DOI 10.1007/s11136-010-9649-x
21. Attkisson CC, Zwick R. The Client Satisfaction Questionnaire: Psychometric properties and correlations with service utilization and psychotherapy outcome. Evaluation program planning. 1982;5(3):233-7. https://doi.org/10.1016/0149-7189(82)90074-X
22. Rahman A, Iqbal Z, Waheed W, et al. Translation and cultural adaptation of health questionnaires. Pakistan Medical Association. 2003;53(4):142-6. https://www.jpma.org.pk/article-details/115?article_id=115
23. Platt S, Bille-Brahe U, Kerkhof A, et al. Parasiticide in Europe: the WHO/EURO multicentre study on parasuicide. I. Introduction and preliminary analysis for 1989. Acta Psychiatrica Scandinavica. 1992;85(2):97-104. https://doi.org/10.1111/j.1600-0447.1992.tb01451.x
24. Schmidt U, Davidson K. Life after self-harm: A guide to the future: Routledge; 2004.
25. Schmidt U, Taylor L, Simic M. Cutting Down: A CBT workbook for treating young people who self-harm: Routledge; 2015.
26. Beecham J, Knapp M. Costing psychiatric interventions. Measuring mental health needs. 2001;2:200-24. https://www.pssru.ac.uk/pub/dp1536.pdf
27. Brooks R, Group E. EuroQol: the current state of play. Health Policy & Planning. 1996;37(1):53-72. https://doi.org/10.1016/0168-8510(96)00822-6.
28. Guest G, Bunce A, Johnson L. How many interviews are enough? An experiment with data saturation and variability. Field methods. 2006;18(1):59-82.https://doi.org/10.1177/1525822X05279903
29. Kuzel AJ. Sampling in qualitative inquiry. 1992. https://psycnet.apa.org/record/1992-97742-002
30. Zerva C, Nghiem M-Q, Nguyen NT, et al. Cited text span identification for scientific summarisation using pre-trained encoders. Scientometrics. 2020;125(3):3109-37. https://doi.org/10.1007/s11192-020-03455-z
31. Braun V, Clarke V, Terry G. Thematic analysis. Qual Res Clin Health Psychol: 95–114. 2014.
32. Fletcher AJ. Applying critical realism in qualitative research: methodology meets method. International Journal of Social Reserach Methodology. 2017;20(2):181-94. https://doi.org/10.1080/13645579.2016.1144401

For peer review only - http://bmjopen.bmj.com/site/about/guidelines.xhtml

33. Hampson L, Williamson P, Wilby M, et al. Recruitment progression rules for internal pilot studies monitoring recruitment. 2015;16(2):1-.https://doi:10.1186/1745-6215-16-S2-O89
34. Naz A, Naureen A, Kiran T, et al. Exploring lived experiences of adolescents presenting with self-harm and their views about suicide prevention strategies: a qualitative approach. International journal of environmental research and public Health (Elsevier). 2021;18(9):4694. https://doi.org/10.3390/ijerph18094694

For peer review only

For peer review only - http://bmjopen.bmj.com/site/about/guidelines.xhtml

Figure:1
Flow Diagram of the YCMAP study

For peer review only

For peer review only - http://bmjopen.bmj.com/site/about/guidelines.xhtml

Figure 2
Theory of Change

For peer review only

For peer review only - http://bmjopen.bmj.com/site/about/guidelines.xhtml

Figure 1
Flow diagram of the YCMAP study

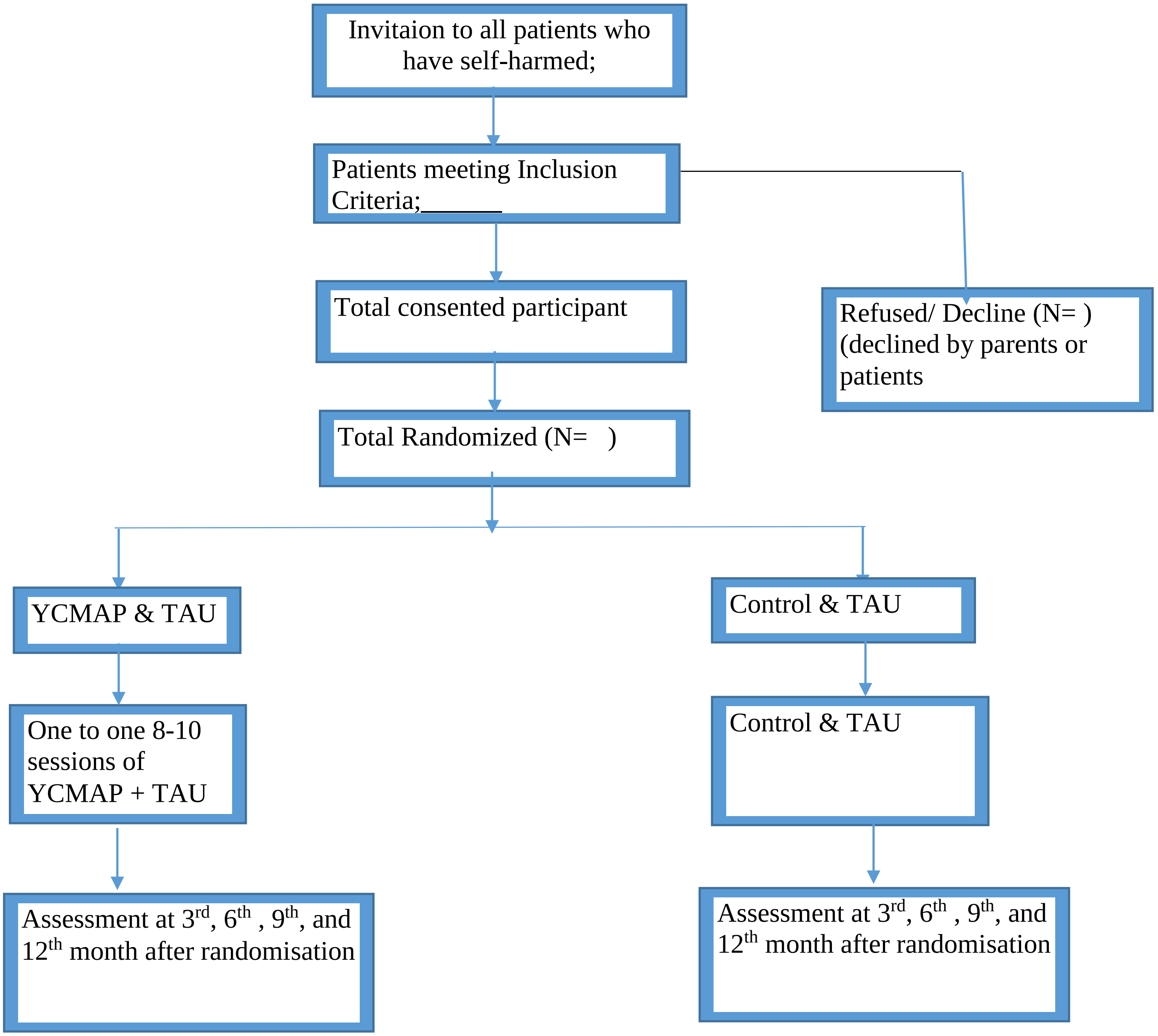

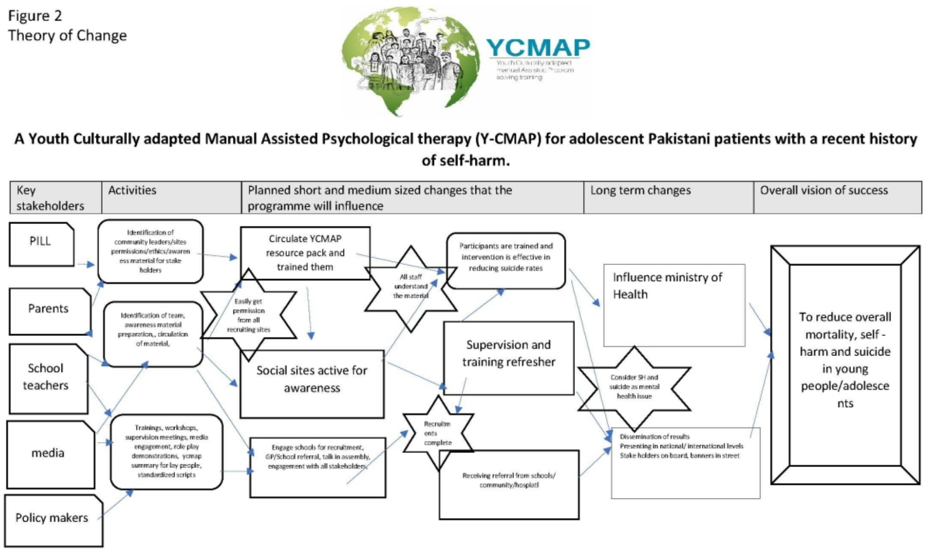

Theory of Change

297x210mm (200 x 200 DPI)

CONSORT 2010 checklist of information to include when reporting a randomised trial*

Section/Topic	Item No	Checklist item	Reported on page No
**Title and abstract**			
	1a	Identification as a randomised trial in the title	1
	1b	Structured summary of trial design, methods, results, and conclusions (for specific guidance see CONSORT for abstracts)	3
**Introduction**			
Background and objectives	2a	Scientific background and explanation of rationale	4
	2b	Specific objectives or hypotheses	5
**Methods**			
Trial design	3a	Description of trial design (such as parallel, factorial) including allocation ratio	7
	3b	Important changes to methods after trial commencement (such as eligibility criteria), with reasons	No change
Participants	4a	Eligibility criteria for participants	7
	4b	Settings and locations where the data were collected	7
Interventions	5	The interventions for each group with sufficient details to allow replication, including how and when they were actually administered	8
Outcomes	6a	Completely defined pre-specified primary and secondary outcome measures, including how and when they were assessed	5-6
	6b	Any changes to trial outcomes after the trial commenced, with reasons	No
Sample size	7a	How sample size was determined	7-8
	7b	When applicable, explanation of any interim analyses and stopping guidelines	No
Randomisation:			
Sequence generation	8a	Method used to generate the random allocation sequence	8
	8b	Type of randomisation; details of any restriction (such as blocking and block size)	8
Allocation concealment mechanism	9	Mechanism used to implement the random allocation sequence (such as sequentially numbered containers), describing any steps taken to conceal the sequence until interventions were assigned	8
Implementation	10	Who generated the random allocation sequence, who enrolled participants, and who assigned participants to interventions	8
Blinding	11a	If done, who was blinded after assignment to interventions (for example, participants, care providers, those	8

For peer review only - http://bmjopen.bmj.com/site/about/guidelines.xhtml

		assessing outcomes) and how	
	11b	If relevant, description of the similarity of interventions	No
Statistical methods	12a	Statistical methods used to compare groups for primary and secondary outcomes	10
	12b	Methods for additional analyses, such as subgroup analyses and adjusted analyses	10
**Results**			
Participant flow (a diagram is strongly recommended)	13a	For each group, the numbers of participants who were randomly assigned, received intended treatment, and were analysed for the primary outcome	No, as it's a protocol
	13b	For each group, losses and exclusions after randomisation, together with reasons	No
Recruitment	14a	Dates defining the periods of recruitment and follow-up	No
	14b	Why the trial ended or was stopped	No
Baseline data	15	A table showing baseline demographic and clinical characteristics for each group	No
Numbers analysed	16	For each group, number of participants (denominator) included in each analysis and whether the analysis was by original assigned groups	No
Outcomes and estimation	17a	For each primary and secondary outcome, results for each group, and the estimated effect size and its precision (such as 95% confidence interval)	No
	17b	For binary outcomes, presentation of both absolute and relative effect sizes is recommended	No
Ancillary analyses	18	Results of any other analyses performed, including subgroup analyses and adjusted analyses, distinguishing pre-specified from exploratory	No
Harms	19	All important harms or unintended effects in each group (for specific guidance see CONSORT for harms)	No
**Discussion**			
Limitations	20	Trial limitations, addressing sources of potential bias, imprecision, and, if relevant, multiplicity of analyses	13
Generalisability	21	Generalisability (external validity, applicability) of the trial findings	13
Interpretation	22	Interpretation consistent with results, balancing benefits and harms, and considering other relevant evidence	13
**Other information**			
Registration	23	Registration number and name of trial registry	3
Protocol	24	Where the full trial protocol can be accessed, if available	Submitting now
Funding	25	Sources of funding and other support (such as supply of drugs), role of funders	14

*We strongly recommend reading this statement in conjunction with the CONSORT 2010 Explanation and Elaboration for important clarifications on all the items. If relevant, we also recommend reading CONSORT extensions for cluster randomised trials, non-inferiority and equivalence trials, non-pharmacological treatments, herbal interventions, and pragmatic trials. Additional extensions are forthcoming: for those and for up to date references relevant to this checklist, see www.consort-statement.org.

For peer review only - http://bmjopen.bmj.com/site/about/guidelines.xhtml

A Youth Culturally adapted Manual Assisted Problem Solving Training (YCMAP) in Pakistani adolescent with a history of self harm: Protocol for multi-centre clinical and cost effectiveness randomised controlled trial.

Journal:	*BMJ Open*
Manuscript ID	bmjopen-2021-056301.R1
Article Type:	Protocol
Date Submitted by the Author:	06-Dec-2021

Complete List of Authors:	Husain, Nusrat; The University of Manchester School of Medical Sciences, Division of Psychology and Mental Health; Manchester Global Foundation<br>Tofique, Sehrish; Pakistan Institute of Living and Learning, Division of Neuro-Cognitive Disorder, Older Adults Mental Health<br>Chaudhry, Imran B.; Greater Manchester West Mental Hlth NHS Fdn Trust; Dr Ziauddin Hospital, Psychiatry<br>Kiran, Tayyeba; Pakistan Institute of Living and Learning, Division of Mood Disorder<br>Taylor, Peter; The University of Manchester, Psychology & Mental Health<br>Williams, Christopher; University of Glasgow, Psychiatry<br>Memon, Rakhshi; University College London, Science and Technology Studies<br>Aggarwal, Shilpa; SRCC Children's Hospital (Narayana Health),, Psychiatry<br>Alvi, Mohsin; Pakistan Institute of Living and Learning, Health Economics<br>Ananiadou, S; The University of Manchester, School of Computer Science<br>Ansari, Moin ; Sir Cowasjee Jehangir Institute Of Psychiatry<br>Aseem, Saadia; The University of Manchester, Division of Psychology and Mental Health<br>Beck, Andrew; Bradford Teaching Hospitals NHS Foundation Trust<br>Alam, Sumira; Services Institute of Medical Sciences, Psychiatry<br>Colucci, Erminia ; Middlesex University,<br>Davidson, Kate; University of Glasgow<br>Edwards, Sarah; University College London, Research Ethics and Governance<br>Emsley, Richard; King's College London, Department of Biostatistics and Health Informatics, Institute of Psychiatry, Psychology and Neuroscience<br>Green, Jonathan; The University of Manchester, Division of Neuroscience &Experimental Psychology<br>Gumber, Anil; Sheffield Hallam University, Health Economics<br>Hawton, Keith; Centre for Suicide Research, Oxford University, Psychiatry<br>Jafri, Farhat; Karachi Medical and Dental College, Community Medicine<br>Khaliq, Ayesha; Pakistan Institute of Living and Learning, Division of Eating Feeding, Nutrition and Elimination Disorders<br>Mason, Thomas; University of Manchester, Division of Population Health, Health Services Research & Primary Care, Manchester Institute for Collaborative Research on Ageing

	Mcreath, Ann; Lifestyle & Wellness<br>Minhas, Ayesha; The Tree House<br>Naeem, Farooq; University of Toronto, Psychiatry<br>Naqvi, Haider; Dow University of Health Sciences, Psychiatry<br>Noureen, Amna; Pakistan Institute of Living and Learning, Division of Child and Adolescent Mental Health<br>Panagioti, Maria; University of Manchester, Division of Population Health, Health Services Research & Primary Care<br>Patel, Anita; Queen Mary University of London,<br>Poppleton, Aaron; The University of Manchester, Center for primary care and health services research,<br>Shiri, Tinevimbo; Liverpool School of Tropical Medicine, Health Economics<br>Simic, Mima; South London and Maudsley NHS Foundation Trust<br>Sultan, Sarwat; Bahauddin Zakariya University, Department of Applied Psychology<br>Nizami, Asad; Institute of Psychiatry, Psychiatry<br>Zadeh, Zainab; Pakistan Institute of Living and Learning, Child and Adolescent Mental health<br>Zafar, Shehla; Iqra University<br>Chaudhry, Nasim; Pakistan Institute of Living and Learning, Research and Development

**Primary Subject Heading**:	Mental health
Secondary Subject Heading:	Public health, Evidence based practice
Keywords:	Suicide & self-harm < PSYCHIATRY, Child & adolescent psychiatry < PSYCHIATRY, MENTAL HEALTH

SCHOLARONE™
Manuscripts

A Youth Culturally adapted Manual Assisted Problem Solving Training (YCMAP) in Pakistani adolescent with a history of self harm: Protocol for multi-centre clinical and cost effectiveness randomised controlled trial.

- Corresponding author (Tayyeba kiran,75600,tayyebakiran@gmail.com, tayyaba.kiran@pill.org.pk)
- Co-authors (full name, dept, institution, city and country)

Prof. Nusrat husain[1] , Division of Psychology and Mental Health, University of Manchester, Manchester, UK
Manchester Global foundation, Manchester, UK
Sehrish Tofique[2] , Division of Neuro-cognitive Disorders, Older Adults Mental Health, Pakistan Institute of Living and learing, Karachi, Pakistan
Prof. Imran Bashir Chaudhry[3] ,Professor of Psychiatry, University of Manchester, Manchester, UK.
Dept of Psychiatry, Ziauddin Hospital, Karachi, Pakistan
Tayyeba Kiran[2] , Division of mood disorder, Pakistan Institute of Living and Learing, Karachi, Pakistan
Dr.Peter Taylor[1] , Division of Psychology and Mental Health, University of Manchester, Manchester, UK
Prof. Christopher Williams[4] ,Professor of Psychosocial Psychiatry , University of Glasgow, Scotland, UK
Rakhshi Memon[5] , Department of Science and Technology Studies, University College London,UK
Dr Shilpa Aggarwal[6], Child and Adolescent Psychiatrist, SRCC Children's Hospital (Narayana Health), Mumbai , India
Mohsin Hassan Alvi[2] , Department of Health Economics,Pakistan Institute of Living and Learing, Karachi, Pakistan
Prof. Sophia Ananiadou[1], School of Computer Science, University of Manchester, Manchester, UK
Prof. Moin Ahmad Ansari[7], Dept of Psychiatry LUMHS, Sir Cowasjee Jehangir Institute of Psychiatry, Karachi, Pakistan
Dr Saadia Aseem[1] , Division of Psychology and Mental Health, University of Manchester, Manchester, UK
Dr Andrew Beck[8], Clinical Psychologist, Head of Clinical Health Psychology, Bradford Teaching Hospitals, NHS Foundation Trust
Dr Sumira Alam[9], Department of Psychiatry,Services Institute Of Medical Sciences, Lahore, Pakistan
Dr Erminia Colucci[10], Dep
artent of Psychology, Middlesex University,London, UK
Prof. Kate Davidson[4], Institute of Health and Wellbeing, University of Glasgow, Scotland, UK
Prof Sarah Edwards[5], Bioethics, University College London, UK
Prof.Richard Emsley[11], Biostatistics & Health Informatics, Institute of Psychiatry, Psychology and Neuroscience,Kings College London, UK
Prof. Jonathan Green[1],Division of Neuroscience &Experimental Psychology,University of Manchester, Manchester, UK
Dr. Anil gumber[12], Principal Health Economist, Sheffield Hallam University, UK
Prof. Keith Hawton[8], Psychiatry, Oxford Health NHS Foundation Trust, University of Oxford, UK
Prof. Farhat Jafri[13], Community Medicine, Karachi Medical and Dental College, Karachi, Pakistan
Ayesha Khaliq[2] , Division of Eating Feeding, Nutrition and Elimination Disorders , Pakistan Institute of Living and Learning, Lahore, Pakistan
Dr. Thomas Mason[1], Division of Population Health, Health Services Research & Primary Care, Manchester Institute for Collaborative Research on Ageing, Manchester, UK
Ann Mcreath[14], Mental Health Nurse, Director at Lifestyle & Wellness, UK
Prof. Ayesha Minhas[15], Psychiatry, The Tree House, Rawalpindi, Pakistan
Prof.Farooq Naeem[16], Psychiatry, University of Toronto, Toronto, Canada
Prof. Haider Ali Naqvi[17], Psychistry, Dow University of Health Sciences, Karachi, Pakistan

For peer review only - http://bmjopen.bmj.com/site/about/guidelines.xhtml

Amna Noureen[2], Division of Child and Adolescents Mental health, Pakistan Institute of Living and Learning, Rawalpindi, Pakistan

Dr. Maria Panagioti[1],Division of Population Health, Health Services Research & Primary Care, Institute for Health Policy and Organisation /Alliance Manchester Business School, Manchester, UK

Prof.Anita Patel[18], Health Economics, Queen Mary, University of London, UK

Dr. Aaron Poppleton[1],NIHR Academic Clinical Fellow in General Practice, Center for Primary Care and Health Services Research,University of Manchester

Tinevimbo Shiri[19], Health Economics, Liverpool School of Tropical Medicine, England, UK

Dr. Mima Simic[20], Child and Adolescent Psychiatrist, The Maudsley Hospital London,NHS foundation England, United Kingdom

Dr. Sarwat Sultan[21], Division of Psychology, Bahauddin Zakaria University, Multan, Pakistan

Prof. Asad Tamizuddin Nizami[22], Psychiatry, Institute of Psychiatry, Rawalpindi, Pakistan

Prof. Zainab Zadeh[2] , Division of Child and Adolescent health, Pakistan Institute of Living and Learning, Karachi, Pakistan

Prof. Shehla Naeem Zafar[23], Community and Public Health Nursing, Iqra University, Karachi, Pakistan

Prof. Nasim Chaudhry[2] , Research and Development, Pakistan Institute of Living and Learning, Karachi, Pakistan

- Word count (excluding title page, abstract, references, figures, tables-4518/4000)
- Start & End Date of Study: March 2019 – Feburary 2023
- Protocol version V2-19th Nov’2021

For peer review only

For peer review only - http://bmjopen.bmj.com/site/about/guidelines.xhtml

ABSTRACT

Introduction: Suicide is a global health concern. Socio-cultural factors have an impact on self-harm and suicide rates. In Pakistan both self-harm and suicide are considered as criminal offense's and are condemned on both religious and social grounds. The proposed intervention "Youth Culturally Adapted Manual Assisted Problem Solving Training (YCMAP)" is based on principles of problem-solving and cognitive behavioural therapy (CBT). YCMAP is a brief, culturally relevant, scalable intervention that can be implemented in routine clinical practice if found to be effective.

Method and Analysis: A multi-centre rater blind randomized control trial (RCT) to evaluate the clinical and cost effectiveness of YCMAP including a sample of 652 participants, aged 12-18 years, presenting to general physicians/clinicians, emergency room after self harm or self referrals. We will test the effectiveness of 8 to 10 individual sessions of YCMAP delivered over three months compared to Treatment as Usual (TAU). Primary outcome measure is repetition of self-harm at 12 months. The seconday outcomes include reduction in suicidal ideation, hopelessness and disress and improvement in health related quality of life. Assessments will be completed at baseline, 3, 6, 9 and 12 month post randomization. The nested qualitative component will explore perceptions about management of self-harm and suicide prevention amongst adolescents and investigate participants' experiences with YCMAP. The study will be guided by the Theory of Change approach to ensure that the whole trial is centred around needs of the end beneficiaries as key stakeholders in the process.

Ethics and Dissemination: Ethics approval has been obtained from the Ethics Committee of University of Manchester, the National Bioethics Committee in Pakistan. The findings of this study will be disseminated through community workshops, social media, conference presentations and peer-reviewed journals.

Trial Registration Number: NCT04131179

For peer review only - http://bmjopen.bmj.com/site/about/guidelines.xhtml

STRENGTHS AND LIMITATIONS OF THIS STUDY

1. This is the first multi centre RCT to evaluate clinical and cost effectiveness of a culturally relevant psychological intervention for young people presenting with self-harm in a Lower and Middle Income Country (LMIC).
2. This trial fulfills an important clinical need of LMICs, specifically in Pakistan.
3. The trial will provide evidence based outcome data to inform and support policy makers in formulating policies for prevention of self-harm and suicide.
4. Stigma, fear of persecution by authorities, peer pressure and educational commitments may act as a barriers to young people participating in the study.
5. The Theory of Change approach will help mitigate some of the risks around stigma and refusal to participate by involving parents and young people from the inception of the trial.

INTRODUCTION

Suicide is one of the major public health concerns globally with 800,000 suicides each year across the globe (1). More than 75% of suicides occur in Low and Middle Income Countries (LMICs). Overall, suicide is the 2nd leading cause of death in individuals between 15-29 years old (1). A recent review reported suicide rates in South Asia to be higher than the global average(2). However, such figures are likely to be an underestimate due to a lack of accurate data on suicide in Pakistan (3) and in many other LMICs (4).

In Pakistan, a conservative Islamic state, policies are changing but suicide and self-harm (defined below) remain criminal acts and are condemned both socially and religiously as a moral wrong. Suicide and self-harm are often considered taboo subjects across the country, thus contributing to a lack of evidence and under-reporting (2). There is, however, accumulating evidence that both self-harm and suicide rates have been increasing in Pakistan but there continues to be notable gaps in evidence(2). In a systematic review of mental health studies of adolescents in India, it was found that self-harm is particularly problematic in young people, with three months prevalence ranging from 3.9% to 25.4% in community based studies (5, 6). Self-harm is one of the strongest predictors of death by suicide (7). Self-harm also carries a substantial economic impact and has been associated with large treatment costs in Pakistan (8). Adolescents with a history of self-harm are at a higher risk of repeating this later in life (9)thus there is an important need to develop effective interventions in order to avoid long-term burden.

In adolescents, self-harm is the result of a highly complex interplay between genetic, biological, psychological, social, and cultural factors (9). Common set of risk factors and psycho-social mechanisms for self-harm within LMICs include being female, experiencing interpersonal

For peer review only - http://bmjopen.bmj.com/site/about/guidelines.xhtml

For peer review only

conflict, suffering from abuse (including domestic/family/gender based violence), hopelessness, and being diagnosed with psychiatric disorder (2). Other psychological influences include feelings of entrapment, a lack of belonging or connection (10). Research in Pakistan is imperative to explore the effectiveness of interventions for potential risk factors to reduce rates of self-harm and suicide (11). The World Health Organization (WHO) Report "preventing Suicide: A Global imperative" recommends "two-fold" public health approach for suicide prevention i.e. to identify the issues, and provide treatment for high-risk individuals.Considering the benefits of adult individual Cognitive Behaviour Therapy (CBT)-based psychotherapy(12), tested in our previous trial in Pakistan with positive results (13) and replicated in our recently completed trial with 901 adults participants in which we had to exclude 265 adolescents (less than 18 years) (CMAP2) (14). An unmet need was recognized to develop CMAP further for adolescent and adapting the similar brief psychological intervention for children and adolescents.Psychological therapies that work in western culture cannot always be implemented in a different culture and adaptation is considered to be essential (15, 16)

Since interpersonal conflicts with family members are a commonly reported precipitant of self-harm episodes in Pakistan (2, 11). It has been reported that problem-solving therapy "Culturally adapted Manual Assisted brief Psychological intervention (CMAP)" may be a useful intervention for prevention of self-harm in Pakistan (11). This psychological intervention, using variants of CBT that are age appropriate for the local culture and customs in Pakistan, may prove to be beneficial in reducing self-harm in adolescents (13). Before this intervention can be implemented at a larger scale, there is a need for establishing its clinical and cost-effectiveness via the proposed trial. We cannot assume that an intervention developed for adults would be suitable for children and adolescents and since self-harm remains a major problem in young people, this planned trial fills an important gap and clinical need.

METHODS AND ANALYSIS

Objectives

This study includes both quantitative and qualitative aspects.

The objectives of the quantitative component are:

1. To determine the clinical effectiveness of YCMAP over 12 months based on the following outcomes:
    a. Primary outcome:
        i. To measure the effectiveness of YCMAP in comparison to treatment as usual (TAU) in terms of repetition of self-harm 12-month post

For peer review only - http://bmjopen.bmj.com/site/about/guidelines.xhtml

randomization. This will be assessed using adapted Suicide Attempt Self-Injury Interview SASII (17).

b. Secondary outcomes:
    i. Suicidal ideation will be assessed using the “Beck Scale for Suicidal ideation (BSI)”(18).
    ii. Feelings of hopelessness will be assessed using the “Beck Hopelessness Scale (BHS)”(19).
    iii. Level of distress will be assessed using a 10-item scale the “Kessler Psychological Distress Scale (K10)”(20).
    iv. EQ-5D- Y will be used to assess “health related quality of Life” (21).
    v. Client Satisfaction Questionnaire-“CSQ-8” will be used to assess participants’ satisfaction with services (22).

Translated versions of all these scales have been developed and used in previous trials CMAP1 (13) and CMAP 2 (11) by following established protocols for translating such measures (23).The scales which were not available in Urdu were translated for an MPhil project (Brief psychological intervention for adolescents who self harm, IPP/BU/ER/103/1377) and reviewed in initial Patient and Public Involvement and Engagement meetings.

Assessments will be carried out at baseline, completion of the intervention (3 months), 6, 9 and 12 months after randomisation. See figure 1 below

2. To determine the cost effectiveness of the YCMAP intervention over 12 months.

The objectives of the qualitative component are:

1. To explore the experience of participants with YCMAP and their perception about benefits and negative or adverse consequence of it.
2. To explore in detail participants’ reasons for continuing (completer) or not continuing (drop out) with the study.
3. To explore clinicians and other stakeholder views (such as school teachers) on the YCMAP and its impact.
4. To explore therapists’ perspective on the delivery of the YCMAP.

The expected outputs of the study are:
a) A manual-assisted evidence based intervention - the YCMAP, ready for integration into the health care services.

For peer review only - http://bmjopen.bmj.com/site/about/guidelines.xhtml

b) Standardized training and supervision package - a resource pack and training program on 'how to do it' for use across health services.

c) Information Tool-kit for the families and the wider community. This will be service-user defined and will be used to increase awareness about mental health in general and self-harm and suicide in young people in particular.

Inclusion Criteria

Adolescets meeting the study inclusion criteria will be invited to take part in the study by their primary care clinician, ward or emergency room (ER) clinician who is making the initial assessment. In the context of this study, self-harm is defined as: *"an act with non-fatal outcome, in which an individual deliberately initiates a non-habitual behaviour that, without interventions from others, will cause self-harm, or deliberately ingests a substance in excess of the prescribed or generally recognised therapeutic dosage, and which is aimed at realizing changes which the subject desired via the actual or expected physical consequences"(24).*

- Age: 12-18 years.
- History of recent self-harm which is defined as " self-harm occurring within the last 3 months (from the initial identification of a potential participant)". This period is considered as high risk for repetition in young people (9).
- Resident in the trial catchment area.
- All participants known to clinical or health services. This will be necessary to ensure all participants have access to TAU.

Exclusion Criteria

- Patients with a severe mental illness such as schizophrenia spectrum and other psychotic disorders, bipolar and severe depressive disorder will be excluded.
- Patients with conditions limiting engagement with assessment/intervention.
- Temporary resident unlikely to be available for follow up.

Design

A multi-centre rater blind RCT with randomisation by individual participants in order to compare the YCMAP in addition to TAU with TAU alone.

Study Site and Population

For peer review only - http://bmjopen.bmj.com/site/about/guidelines.xhtml

Study sites are all participating primary care clinics,emergency departments and medical wards of general hospitals in five major cities of Pakistan, Karachi, Hyderabad, Lahore, Multan and Rawalpindi.

Sample Size

The sample size is based on the primary outcome, repetition of self-harm in a 12-month period (yes/no). The TAU arm of the study has an expected self-harm rate of 20% (5). A clinically important effect would be a reduction to 7.5% in the intervention group. Under these presumptions, and assuming a 5% significance level and 90% power, a study with no clustering would require 158 patients per arm. The study has a partially nested design due to therapist clustering in the YCMAP arm. Our sample size calculation has taken account of this by adjusting the sample size upwards, conservatively assuming clustering in both arms. Based on previous analysis of therapist trials, we believe that the intraclass correlation coefficient (ICC) is likely to have a value between 0.01 and 0.05 for this type of outcome measure. Assuming an ICC of 0.05, and a cluster size of 16 patients per therapist, a design effect of 1.75 is calculated. This increases the numbers required to 277 per arm. Furthermore,based on our previous work there is expected to be a 15% loss to follow-up, and so the final numbers recruited will be 326 per arm, with a total sample of 652 participants.

Randomisation

The researcher will complete a checklist, confirming eligibility, obtain written (or verbal audio-recorded consent from the subject due to current pandemic COVID-19 SOPs), and complete the baseline measures. The researcher will contact the randomisation centre, who will re-check eligibility, record baseline measures and assign a participant's trial number. Treatment assignment will then be determined using stochastic minimisation controlling for gender, age and type of self-harm behaviour. The independent statistician would inform the project manager of the random assignments, but this information would not be shared with research staff, so that they remain unaware of whether a particular participant receives YCMAP or not. Participants assigned to the YCMAP would then be contacted by a therapist, who will arrange an initial meeting within 2-4 weeks after baseline to start the intervention. Participants in the TAU group would be informed of their allocation to this group after randomisation had taken place.

Recruitment and Baseline Assessment

Potentially eligible individuals will be identified by clinical staff at the participating recruitment sites. An age appropriate version of the Participant Information Sheet (PIS) will be provided alongside the standard PIS, so both the parent/guardian and child can access this information. For

For peer review only - http://bmjopen.bmj.com/site/about/guidelines.xhtml

individuals who cannot read, information about the study will be summarised by the clinician for them. The consent was obtained from the parent/guardian along with consent from the participating young person (The Nuffield Council for Bioethics guidance suggests that consent rather than assent should be sought from young people in research).Therefore, it is ensured and clearly mentioned in the participant information sheet for both the young people and their parents that it is mandatory for parents/guardians to consent for young person to participate in the study.

Intervention

YCMAP is a "Youth culturally adapted manual assisted psychological intervention" based on CBT principles. It comprises of 8-10 sessions delivered over three months.The first eight sessions are offered weekly and further sessions fortnightly on a one to one basis and each session lasts for about 60 minutes. The YCMAP has been culturally adapted with permission from "CMAP" (15), "Life after self-harm" (25) and "Cutting down: A CBT workbook for treating young people who self-harm"(26). The intervention includes psycho-education and a comprehensive cognitive behavioural assessment of the self-harm attempt using virtual stories of four young people. The therapy focuses on current problems that contributed to the self-harm episode. Therapists and adolescent clients choose from a list of techniques those which are most relevant to the client's problems. Therapy is therefore adapted to fit with the clients problems and primarily utilises problem solving, CBT, and dialectical therapy strategies to bring about change.

To help determine the most appropriate coping strategy a coping tree is designed. Training in assertiveness and anger management are offered to help the young person to develop resilience to cope with stress. To the existing YCMAP we will also aim to review the look and feel of the resource by including licenced content from My Big Life - a course developed by our collaborative partner Five Areas Limited. This accessible, story-based approach uses illustrations of young people facing different scenarios at home and at school to illustrate key CBT-based concepts: Understanding your feelings -How to get a Big Life (behavioural activation) - Thinking in a Big Life way (identifying and changing thoughts that upset and affect how you feel) - Relaxation approaches - Building inner confidence - Practice scenarios - Trainer notes and linked worksheets/prompt cards and posters. Asian versions of the course already exist with amended artwork. As part of the development programme selected content will be added to the course and trainer notes will be modified (based on feedback) as needed.

All research staff will be required to have at least an undergraduate level degree in a relevant area (e.g. psychology) and will be trained in "Good Clinical Practice (GCP) " in research, consent process, and use of the assessment measures. Regular supervision meetings will be arranged for research staff and therapists for case discussions, identifying and managing distress in participants. A study protocol will be in place prior to the start of the trial. This will include details about how to manage difficult situations arising in research, safety and lone working arrangements.

For peer review only - http://bmjopen.bmj.com/site/about/guidelines.xhtml

Treatment as Usual (TAU)

Local psychiatric, medical and primary care services provide standard routine care according to their clinical judgment and available resources. We will administer Client Services Receipt Interview (CSRI) (27) to obtain details regarding treatment received by each study participant.These participants will receive an initial assessment along with TAU as ascertained by their treating doctor at the hospital or their GP. The current practice is that self-harm patients are not routinely referred to mental health services. Research staff will record the nature and intensity of the routine care delivered for each participant.

Statistical Analysis

Statistical analysis will be based on intention-to-treat principles, subject to the availability of data. During the course of the trial, periodic random quality checks of data will be carried out by the trial statistician blind to treatment allocation. Once data entry has been completed, preliminary data analysis will be carried out blind to treatment allocation, prior to un-blinding. The results of the trial will follow the standard CONSORT recommendations. Baseline and follow-up data will be summarised using the appropriate descriptive statistics and graphical summaries. Treatment effects will be presented with 95% confidence intervals. We will investigate baseline factors that predict nonresponse using a logistic random effects model as non-response may be clustered by therapy group. The statistical analysis of the primary outcome measure (repetition of the self-harm episode within 12-months) will be based on a logistic random effects model with randomised treatment, age, gender and type of self-harm as fixed effects and a random effect of therapist in the treatment arm, with individuals in the control arm being treated as clusters of size 1. The continuous secondary outcome measures will calculate treatment effects using a linear mixed model, with a random effect of therapist and the set of baseline covariates as above including baseline values of the outcome where available. A single model will be fitted across all time-points, with a fixed effect for time, and interactions between time and treatment group. There will be no adjustment to secondary outcomes CIs for multiple testing. Binary secondary outcomes will take a similar approach to continuous outcomes, but will use logistic random effects models.

Economic Evaluation

The economic evaluation will take a societal perspective that incorporates the costs of both formal and informal health care and other relevant economic impacts such as on education. It will include financial impacts on providers and out of pocket payments by participants and their families. The use and payment for services will be collected using the CSRI (27) with patients and their families at baseline and at each follow up assessment. The use of the YCMAP intervention will be recorded

For peer review only - http://bmjopen.bmj.com/site/about/guidelines.xhtml

separately by the trial team. Appropriate unit costs will be collected and attached to individual-level resource use quanitities to estimate total care costs for each individual. Cost estimates for the YCMAP intervention will include a) the salary of the therapists and b) indirect costs like session preparation, supervision, on-costs and capital overheads. Comparisons of total costs between the YCMAP and TAU arms will be based on non-parametric bootstrapped regressions (with co-variates for baseline costs, outcome measures and other key baseline factors) to account for the likely non-normal distribution in costs. An inital analysis will examine whether any additional costs associated with the YCMAP intervention are offset by savings elsewhere.

More formal assessments of cost-effectiveness will link between-arm differences in average costs with between-arm differences in a) the primary outcome measure, repetition of self-harm (SASII) and b) quality adjusted life year (QALY) gains. QALYs will be estimated by applying relevant utility weights to health states measured through EQ-5D-Y (21, 28). Costs and outcomes will be linked in the form of incremental cost-effectiveness ratios (ICERs) where relevant, and cost-effectiveness acceptability curves (CEACs) based on the net benefit approach. As per the clinical effectiveness analyses, we will use an intention-to-treat approach for all economic analyses.

Qualitative Data Analysis

A purposefully selected subset (stratified by age, gender and self-harm severity) of participants in the treatment group will be invited to complete a qualitative one-to-one digitally recorded interview (face to face or via phonecall) lasting 1-1.5 hours. This will focus on their experience with the YCMAP intervention, the barriers and facilitators to engagement and perceived positive or negative experience with the intervention. A sample of 12-20 participants is likely to be sufficient to ensure data saturation (29) (30). Focus groups ($k = 2$ with 8-10 participants each) with therapists and key stakeholders will also be conducted to enable a further process analysis concerning the wider implementation of YCMAP into the Pakistani health systems. During both the individual and group interviews, broader suggestions for management and prevention of self-harm among young Pakistanis will also be collected to inform future service and research development.

We will explore perspectives of therapists and their supervisors about training and delivery of the model of care. We will also interview other key stakeholders to establish facilitators and barriers to implementation of the intervention in Pakistan.Targeted interviews will be done with a sample of participants who completed the intervention and a sample of participants who 'dropped out' before completion. Up to 30 interviews are likely to be needed with patient participants, to achieve category saturation. New text mining tools will be used to link heterogeneous data, to ensure that knowledge scattered with in lengthly textual data reaches clinicians, patients and policy makers to support decision making using different sources of information (31). Qualitative data will be analysed using Thematic Analysis (32) adopting a critical realist perspective (33).This will enable

For peer review only - http://bmjopen.bmj.com/site/about/guidelines.xhtml

the research team to draw conclusions on the intervention whilst accounting for contextual and cultural factors.

Topic guides will be developed through discussion amongst trained qualitative researchers as well as with reference to relevant literature. Semi- structured interviews will be done to explore views on the "effectiveness and sustainability" of the YCMAP intervention in the management of people presenting with self-harm. Written or verbal consent at the time of interview (face to face or telephone) or prior to the interview (considering current pandemic) will be obtained from study participants and stake holders.

Internal pilot study

We have included an internal pilot phase (initial 12 months) with clear stop/go criteria across the study centres. This is included to ensure achievement of recruitment targets is viable across all sites included in this full trial. Progression to the full trial will be dependent on meeting the key objectives and stop/go criteria outlined below. The pilot phase will examine the processes for recruitment and logistical practices to support the effective execution of the full RCT. The internal pilot phase will be conducted in line with the National Institute of Health Research (NIHR) guidelines (34). The key objectives of the pilot are as follows; i) Recruitment of sufficient number of General Practitioners/Hospital Departments to support the trial. ii) Identify the proportion of eligible participants recruited across the study centres, and examine the feasibility of achieving recruitment targets to a full multicenter RCT. The pilot will be judged successful if sufficient GPs and hospitals are recruited to enroll 200 participants in the first year of recruitment i.e 4-12 months.We will monitor recruitment and identify the reasons for any shortfall on a monthly basis. The predetermined Stop-Go criteria is as follows:

i. Go if successfully recruit 180 (target minus 10%) or more participants during pilot study phase

ii. Rescue plan to be implemented if less than 180 but greater than 120 participants recruited in pilot phase.

iii. Stop if less than 120 participants recruited into the trial.

Theory of Change (ToC)

The study team uses ToC as the standard framework for all research projects. The overall study will be underpinned by the ToC causal model of planning, monitoring, evaluation and impact assessment to ensure that marginalised voices are included in developing the vision, identifying barriers and challenges from the lens of the end beneficiaries and short, medium and long term outcomes are developed and delivered so real change happens from the perspective of the target

For peer review only - http://bmjopen.bmj.com/site/about/guidelines.xhtml

group. A ToC workshop with key stakeholders has already taken place to ensure all stakeholders have a buy in and ownership of the process. See Figure 2

The ToC causal diagram as an output is evidence of stakeholder engagement and ownership. Also,ToC will enable us to show how trial results are adopted in wider practice and on what basis,what barriers and challenges were faced during the trial and what assumptions were made in defining the goal statements? Informed by the ToC workshop, a Young People's Advisory Group will be set up to provide insight into the lived experiences of today's youth in Pakistan.

As religiosity was a dominant thread from the community engagement exercise, there was recognition that spiritual advice was often the first point of contact for patients rather than health professionals. The trial was not designed to try to replace such religious norms but to work alongside them while recognising that there could be delays in referrals. There was also no restriction on accessing spiritual guidance making professional psychological therapies an addition not a substitute for such support. Importantly, mental health concerns are stigmatising and suicide is still considered as a criminal offense.

Working with religious leaders was therefore a critical part of community engagement with Y-CMAP and its evaluation through the RCT the concept of 'dawa and dua' medicine and prayer going hand in hand as part of therapy tend to promote adherence and engagement.

COVID-19 Continuity Plan

Due to global pandemic COVID-19, amendments to study procedures in response to the lock down and social distancing in Pakistan were made to ensure smooth running of the project. See supplementary file.

Patient and Public Involvement and Engagement (PPIE) Group

This group will help to ensure that the research agenda for self-harm and suicide prevention studies is informed by and aligned with service users and carer priorities.

Trial Steering Committee (TSC)

We will convene an independently chaired TSC to approve and provide oversight of the trial throughout its various stages. The TSC will include the chair, user representative, three PIs, an independent statistician and a representative from the local health department. This will be independent of the trial management team. The (TSC) will convene annually, but twice in the first year, with feedback from the Chair as and when needed.

For peer review only - http://bmjopen.bmj.com/site/about/guidelines.xhtml

Data Monitoring and Ethics Committee (DMEC)

The DMEC will oversee the data and advise the TSC on any ethical or safety concerns.

Ethics

Ethics approval for the trial has been obtained from the Research Ethics Committee of University of Manchester (Ref: 2019-5024-10755) and the National Bioethics Committee in Pakistan (Ref: No.4-87/NBC-419/19/1213).

Dissemination

The study findings will be disseminated using social media, at national and international conferences in partnership with the "service user", "carer" and "community organizations". Also, study results will be submitted to peer and non peer-reviewed journals for publication.

Discussion

There has been a recent significant increase in number of trials related to self-harm and suicide prevention which reflects the need and international concern about self-harm/suicide prevention. However, in Pakistan evidence based research is limited with no specific intervention for adolescents with self harm history. The evidence suggests that CMAP intervention for adults is effective in Pakistan (35). The components of CMAP intervention may also be beneficial for adolescents presenting after self-harm, therefore there is a strong need to determine the effectiveness of culturally adapted manual assisted problem solving intervention for adolescents in Pakistan. Findings will also contribute to evidence based treatments for self-harm and will meet important clinical needs of Pakistan and will help guide policy makers in developing suicide prevention policies.

Authors Contributions

NH, NC, TK, IBC and ST involved in conceptualisation, designing and planning of the project (including protocol development). EC, SA reviewed qualitative component. ZZ, CW, PT, KD and FN reviewed intervention section, RM facilitated the ToC process. MA, RE reviewed statistical analysis plan and AP, AG reviewed economic evaluation plan. All authors SA, SoA, MHA, AB, SuA, SE, JG, KH, FJ, AK, TM, AnMc, AM, HAN, AN, MP, AaP, TS, MS, SS, ATN, and SNZ gave their valuable inputs in finalizing the study protocol.

Funding Statement

The Medical research Council /DFID/NIHR programme (MR/R022461/1) has funded this trial.

For peer review only - http://bmjopen.bmj.com/site/about/guidelines.xhtml

The funder has no role in designing the study methodology, data analysis and interpretation, manuscript writing and in the decision to submit the protocol for publication.

Competing Interests

All authors have read and declared that we have no competing interests with the exception of NH, NC, IBC, TK and CW. NH, former Trustee of "Pakistan Institute of Living and Learning (PILL)", "Abaseen Foundation (UK)" and "Lancashire Mind (UK)". At "Manchester Global Foundation", he is the Chair of Board of Trustees. He is also a member of the executive committee for the Faculty of Academic Psychiatry, at the Royal College of Psychiatrists. NH is a NIHR Senior Investigator. NC is Associate Director of Global Mental Health and Cultural Psychiatry Research Group. IBC, former Trustee of "PILL" is Honorary Professor at the University of Manchester. NH, IBC and NC have received support for educational programs and/or travel support and/or speaker fees from pharmaceutical companies. CW is the author of a book aimed at suicide prevention, and has written a range of books and online CBT-based course resources that are available as both, free access and on a commercial basis. NH, NC, IBC and TK's time is partially funded by the Global Challenges Research Fund "South Asia Harm Reduction Movement-SAHAR M" (MR/P028144/1).

Acknowledgement

We express our gratitude to all team members, researchers, consultants and investigators for their support and advice in the development and setting up of the trial. We would like to thank community gate keepers, service uers and patient and public involvement group members for their kind support, input and feedback on the development of the funding application, spreading awareness among masses and in trial recruitments.

We would also like to thank Trial Steering Committee (TSC) members, Prof. David Kingdon (Chair), Dr. Mehmood Shaukat, Faraz Usman and Data Management and Ethics Committee (DMEC) members Prof. Unaiza Niaz (Chair), Dr. Qamar Saeed and Dr. Maryum Ilyas for overseeing the trial's progress and management.

For peer review only - http://bmjopen.bmj.com/site/about/guidelines.xhtml

References:

1. WorldHealthOrganization. Preventing suicide: A global imperative: World Health Organization; 2014.
2. Shekhani SS, Perveen S, Akbar K, et al. Suicide and deliberate self-harm in Pakistan: a scoping review. BMC psychiatry. 2018;18(1):1-15.
3. Farooq U, Majeed M, Bhatti JA, et al. Differences in reporting of violence and deliberate self harm related injuries to health and police authorities, Rawalpindi, Pakistan. 2010;5(2).
4. Jordans MJ, Kaufman A, Brenman NF, et al. Suicide in South Asia: a scoping review. 2014;14(1):358.
5. Aggarwal S, Berk M. Evolution of adolescent mental health in a rapidly changing socioeconomic environment: A review of mental health studies in adolescents in India over last 10 years. J Asian journal of psychiatry
2015;13:3-12.
6. Aggarwal S. Suicide in India. J British medical bulletin. 2015;114(1).
7. Ribeiro J, Franklin J, Fox KR, et al. Self-injurious thoughts and behaviors as risk factors for future suicide ideation, attempts, and death: a meta-analysis of longitudinal studies. 2016;46(2):225-36.
8. Shahid M, Khan MM, Naqvi H, et al. Cost of treatment of deliberate self-harm: a study from Pakistan. 2008;29(4):213-5.
9. Hawton K, Saunders KE, O'Connor RC. Self-harm and suicide in adolescents. The Lancet Neurology. 2012;379(9834):2373-82.
10. Klonsky ED, May AM. The three-step theory (3ST): A new theory of suicide rooted in the "ideation-to-action" framework. International Journal of Cognitive Therapy. 2015;8(2):114-29.
11. Husain MO, Umer M, Taylor P, et al. Demographic and psychosocial characteristics of self-harm: The Pakistan perspective. Psychiatry research. 2019;279:201-6.
12. Witt KG, Hawton K, Hetrick S, et al. Interventions for self-harm in children and adolescents. 2020.
13. Husain N, Afsar S, Ara J, et al. Brief psychological intervention after self-harm: randomised controlled trial from Pakistan. The British Journal of Psychiatry. 2014;204(6):462-70.
14. Kiran T, Chaudhry N, Bee P, et al. Clinicians' Perspectives on Self-Harm in Pakistan: A Qualitative Study. Frontiers in psychiatry. 2021;12:484.
15. Degnan A, Baker S, Edge D, et al. The nature and efficacy of culturally-adapted psychosocial interventions for schizophrenia: a systematic review and meta-analysis. Psychological Medicine. 2018;48(5):714-27.
16. Rathod S, Gega L, Degnan A, et al. The current status of culturally adapted mental health interventions: a practice-focused review of meta-analyses. Neuropsychiatric disease treatment. 2018;14:165.
17. Linehan MM, Comtois KA, Brown MZ, et al. Suicide Attempt Self-Injury Interview (SASII): development, reliability, and validity of a scale to assess suicide attempts and intentional self-injury. Psychological assessment. 2006;18(3):303.
18. Beck AT, Steer RA. Manual for the Beck scale for suicide ideation. San Antonio, TX: Psychological Corporation
1991;63.

For peer review only - http://bmjopen.bmj.com/site/about/guidelines.xhtml

19. Beck AT, Steer RA, Pompili M. BHS, Beck hopelessness scale: manual: Psychological corporation San Antonio, TX; 1988.
20. Andrews G, Slade T. Interpreting scores on the Kessler psychological distress scale (K10). Australian and New Zealand journal of public health. 2001;25(6):494-7.
21. Ravens-Sieberer U, Wille N, Badia X, et al. Feasibility, reliability, and validity of the EQ-5D-Y: results from a multinational study. Quality of life research. 2010;19(6):887-97.
22. Attkisson CC, Zwick R. The Client Satisfaction Questionnaire: Psychometric properties and correlations with service utilization and psychotherapy outcome. Evaluation program planning. 1982;5(3):233-7.
23. Rahman A, Iqbal Z, Waheed W, et al. Translation and cultural adaptation of health questionnaires. Pakistan Medical Association. 2003;53(4):142-6.
24. Platt S, Bille-Brahe U, Kerkhof A, et al. Parasiticide in Europe: the WHO/EURO multicentre study on parasuicide. I. Introduction and preliminary analysis for 1989. Acta Psychiatrica Scandinavica. 1992;85(2):97-104.
25. Schmidt U, Davidson K. Life after self-harm: A guide to the future: Routledge; 2004.
26. Schmidt U, Taylor L, Simic M. Cutting Down: A CBT workbook for treating young people who self-harm: Routledge; 2015.
27. Beecham J, Knapp M. Costing psychiatric interventions. Measuring mental health needs. 2001;2:200-24.
28. Brooks R, Group E. EuroQol: the current state of play. Health Policy & Planning. 1996;37(1):53-72.
29. Guest G, Bunce A, Johnson L. How many interviews are enough? An experiment with data saturation and variability. Field methods. 2006;18(1):59-82.
30. Kuzel AJ. Sampling in qualitative inquiry. 1992.
31. Zerva C, Nghiem M-Q, Nguyen NT, et al. Cited text span identification for scientific summarisation using pre-trained encoders. Scientometrics. 2020;125(3):3109-37.
32. Braun V, Clarke V, Terry G. Thematic analysis. Qual Res Clin Health Psychol: 95–114. 2014.
33. Fletcher AJ. Applying critical realism in qualitative research: methodology meets method. International Journal of Social Reserach Methodology. 2017;20(2):181-94.
34. Hampson L, Williamson P, Wilby M, et al. Recruitment progression rules for internal pilot studies monitoring recruitment. 2015;16(2):1-.
35. Naz A, Naureen A, Kiran T, et al. Exploring lived experiences of adolescents presenting with self-harm and their views about suicide prevention strategies: a qualitative approach. International journal of environmental research and public Health (Elsevier). 2021;18(9):4694.

For peer review only

For peer review only - http://bmjopen.bmj.com/site/about/guidelines.xhtml

Figure:1
Flow Diagram of the YCMAP study

For peer review only - http://bmjopen.bmj.com/site/about/guidelines.xhtml

Figure: 2
Theory of Change

For peer review only

For peer review only - http://bmjopen.bmj.com/site/about/guidelines.xhtml

Supplementary File
COVID 19 Continuity plan

For peer review only

For peer review only - http://bmjopen.bmj.com/site/about/guidelines.xhtml

Figure 1
Flow diagram of the YCMAP study

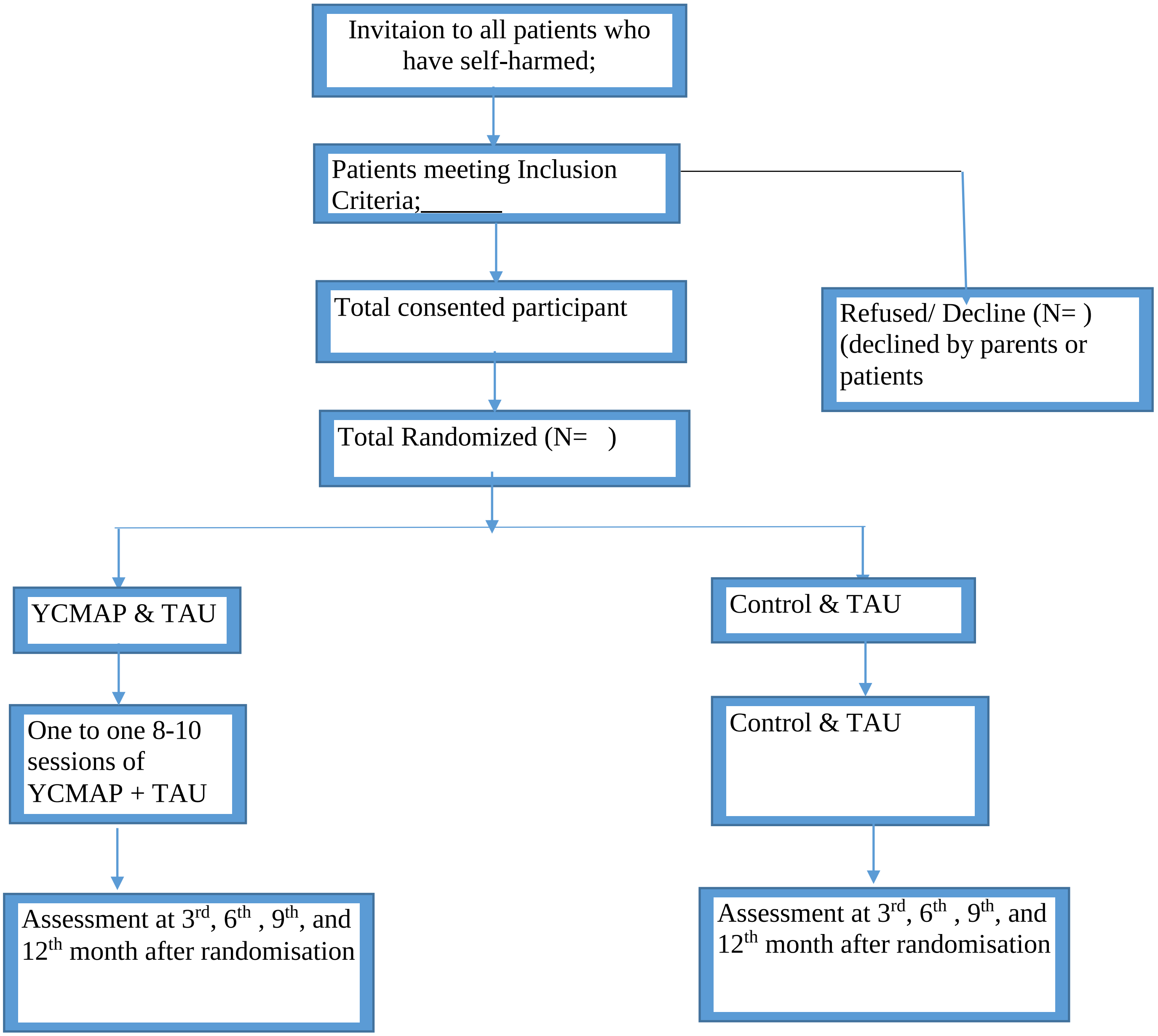

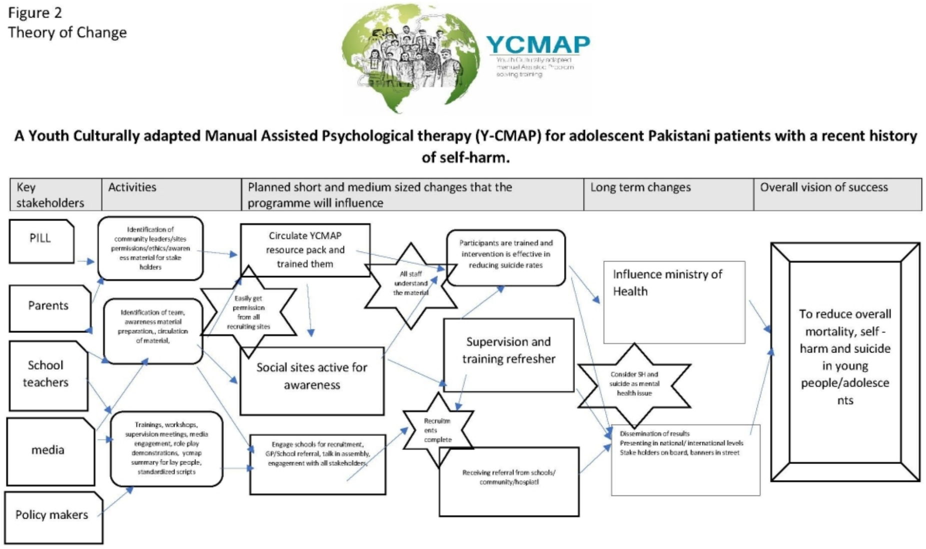

Theory of Change

297x210mm (200 x 200 DPI)

COVID-19 continuity plan

In March 2020, due to the first wave of global pandemic-COVID-19 there was a need to make amendments to study procedures in response to the lock down and social distancing in Pakistan. The trial during this period was in the internal pilot phase and in order to ensure smooth running of the project certain changes were made to continue with collection of data, follow ups and delivery of the intervention.

Follows ups (3rd, 6th, 9th and 12th month after randomization):

Initial ethical approval was obtained for face to face assessment and intervention. However, after COVID-19, we submitted ethics amendment for data collection and delivery of intervention digitally (i.e. through telephone call, zoom, skype or other remote means) so that the study team could continue their work and meet targets on time with working from home condition. The consent form that was previously completed on paper further consents will be obtained remotely and the assessment measures to be completed remotely as well. Recordings of consent will be stored in password protected systems.

YCMAP Intervention:

YCMAP intervention comprises of 8-10 sessions delivered in a face to face setting, usually in research offices, community clinics and homes (as per participant choice). As of 20th march 2020, due to lock down across Pakistan it was difficult to deliver face to face sessions. To comply with preventive measures such as social distancing and lock down guidelines, the YCMAP intervention will be delivered remotely, via a phone call, WhatsApp call, zoom or skype rather than in person face to face sessions. Contingency plan will be implemented to manage confidentiality and safe guarding risks.

Qualitative Interviews:

Qualitative interviews will be completed face to face or through telephone. Due to the pandemic situation COVID-19, topic guides will be revised in order to explore the experience of change in mode of delivery of intervention as well as impact of Covid 19 after discussing it with supervisors (EC, NC).

Supervision**:**

Arrangements for supervision of the therapists will continue as per protocol. The supervisions will continue to be carried out remotely via video link.

For peer review only

Trainings:

Arrangements for training of YCMAP team members will continue and carried out remotely via video link

For peer review only

BMJ Open

A Youth Culturally adapted Manual Assisted Problem Solving Training (YCMAP) in Pakistani adolescent with a history of self harm: Protocol for multi-centre clinical and cost effectiveness randomised controlled trial.

Journal:	*BMJ Open*
Manuscript ID	bmjopen-2021-056301.R2
Article Type:	Protocol
Date Submitted by the Author:	15-Apr-2022

Complete List of Authors:	Husain, Nusrat; The University of Manchester School of Medical Sciences, Division of Psychology and Mental Health; Manchester Global Foundation<br>Tofique, Sehrish; Pakistan Institute of Living and Learning, Division of Neuro-Cognitive Disorder, Older Adults Mental Health<br>Chaudhry, Imran B.; Greater Manchester West Mental Hlth NHS Fdn Trust; Dr Ziauddin Hospital, Psychiatry<br>Kiran, Tayyeba; Pakistan Institute of Living and Learning, Division of Mood Disorder<br>Taylor, Peter; The University of Manchester, Psychology & Mental Health<br>Williams, Christopher; University of Glasgow, Psychiatry<br>Memon, Rakhshi; University College London, Science and Technology Studies<br>Aggarwal, Shilpa; SRCC Children's Hospital (Narayana Health),, Psychiatry<br>Alvi, Mohsin; Pakistan Institute of Living and Learning, Health Economics<br>Ananiadou, S; The University of Manchester, School of Computer Science<br>Ansari, Moin ; Sir Cowasjee Jehangir Institute Of Psychiatry<br>Aseem, Saadia; The University of Manchester, Division of Psychology and Mental Health<br>Beck, Andrew; Bradford Teaching Hospitals NHS Foundation Trust<br>Alam, Sumira; Services Institute of Medical Sciences, Psychiatry<br>Colucci, Erminia ; Middlesex University,<br>Davidson, Kate; University of Glasgow<br>Edwards, Sarah; University College London, Research Ethics and Governance<br>Emsley, Richard; King's College London, Department of Biostatistics and Health Informatics, Institute of Psychiatry, Psychology and Neuroscience<br>Green, Jonathan; The University of Manchester, Division of Neuroscience &Experimental Psychology<br>Gumber, Anil; Sheffield Hallam University, Health Economics<br>Hawton, Keith; Centre for Suicide Research, Oxford University, Psychiatry<br>Jafri, Farhat; Karachi Medical and Dental College, Community Medicine<br>Khaliq, Ayesha; Pakistan Institute of Living and Learning, Division of Eating Feeding, Nutrition and Elimination Disorders<br>Mason, Thomas; University of Manchester, Division of Population Health, Health Services Research & Primary Care, Manchester Institute for Collaborative Research on Ageing

	Mcreath, Ann; Lifestyle & Wellness<br>Minhas, Ayesha; The Tree House<br>Naeem, Farooq; University of Toronto, Psychiatry<br>Naqvi, Haider; Dow University of Health Sciences, Psychiatry<br>Noureen, Amna; Pakistan Institute of Living and Learning, Division of Child and Adolescent Mental Health<br>Panagioti, Maria; University of Manchester, Division of Population Health, Health Services Research & Primary Care<br>Patel, Anita; Queen Mary University of London,<br>Poppleton, Aaron; The University of Manchester, Center for primary care and health services research,<br>Shiri, Tinevimbo; Liverpool School of Tropical Medicine, Health Economics<br>Simic, Mima; South London and Maudsley NHS Foundation Trust<br>Sultan, Sarwat; Bahauddin Zakariya University, Department of Applied Psychology<br>Nizami, Asad; Institute of Psychiatry, Psychiatry<br>Zadeh, Zainab; Pakistan Institute of Living and Learning, Child and Adolescent Mental health<br>Zafar, Shehla; Iqra University<br>Chaudhry, Nasim; Pakistan Institute of Living and Learning, Research and Development
**Primary Subject Heading**:	Mental health
Secondary Subject Heading:	Public health, Evidence based practice
Keywords:	Suicide & self-harm < PSYCHIATRY, Child & adolescent psychiatry < PSYCHIATRY, MENTAL HEALTH

SCHOLARONE™
Manuscripts

A Youth Culturally adapted Manual Assisted Problem Solving Training (YCMAP) in Pakistani adolescent with a history of self-harm: Protocol for multi-centre clinical and cost effectiveness randomised controlled trial.

- Corresponding author (Tayyeba kiran,75600,tayyebakiran@gmail.com, tayyaba.kiran@pill.org.pk)
- Co-authors (full name, dept, institution, city and country)

Prof. Nusrat husain[1] , Division of Psychology and Mental Health, University of Manchester, Manchester, UK
Manchester Global foundation, Manchester, UK
Sehrish Tofique[2] , Division of Neuro-cognitive Disorders, Older Adults Mental Health, Pakistan Institute of Living and learing, Karachi, Pakistan
Prof. Imran Bashir Chaudhry[3] ,Professor of Psychiatry, University of Manchester, Manchester, UK.
Dept of Psychiatry, Ziauddin Hospital, Karachi, Pakistan
Tayyeba Kiran[2] , Division of mood disorder, Pakistan Institute of Living and Learing, Karachi, Pakistan
Dr.Peter Taylor[1] , Division of Psychology and Mental Health, University of Manchester, Manchester, UK
Prof. Christopher Williams[4] ,Professor of Psychosocial Psychiatry , University of Glasgow, Scotland, UK
Rakhshi Memon[5] , Department of Science and Technology Studies, University College London,UK
Dr Shilpa Aggarwal[6], Child and Adolescent Psychiatrist, SRCC Children's Hospital (Narayana Health), Mumbai , India
Mohsin Hassan Alvi[2] , Department of Health Economics,Pakistan Institute of Living and Learing, Karachi, Pakistan
Prof. Sophia Ananiadou[1], School of Computer Science, University of Manchester, Manchester, UK
Prof. Moin Ahmad Ansari[7], Dept of Psychiatry LUMHS, Sir Cowasjee Jehangir Institute of Psychiatry, Karachi, Pakistan
Dr Saadia Aseem[1] , Division of Psychology and Mental Health, University of Manchester, Manchester, UK
Dr Andrew Beck[8], Clinical Psychologist, Head of Clinical Health Psychology, Bradford Teaching Hospitals, NHS Foundation Trust
Dr Sumira Alam[9], Department of Psychiatry,Services Institute Of Medical Sciences, Lahore, Pakistan
Dr Erminia Colucci[10], Departent of Psychology, Middlesex University,London, UK
Prof. Kate Davidson[4], Institute of Health and Wellbeing, University of Glasgow, Scotland, UK
Prof Sarah Edwards[5], Bioethics, University College London, UK
Prof.Richard Emsley[11], Biostatistics & Health Informatics, Institute of Psychiatry, Psychology and Neuroscience,Kings College London, UK
Prof. Jonathan Green[1],Division of Neuroscience &Experimental Psychology,University of Manchester, Manchester, UK
Dr. Anil gumber[12], Principal Health Economist, Sheffield Hallam University, UK
Prof. Keith Hawton[8], Psychiatry, Oxford Health NHS Foundation Trust, University of Oxford, UK
Prof. Farhat Jafri[13], Community Medicine, Karachi Medical and Dental College, Karachi, Pakistan
Ayesha Khaliq[2] , Division of Eating Feeding, Nutrition and Elimination Disorders , Pakistan Institute of Living and Learning, Lahore, Pakistan
Dr. Thomas Mason[1], Division of Population Health, Health Services Research & Primary Care, Manchester Institute for Collaborative Research on Ageing, Manchester, UK
Ann Mcreath[14], Mental Health Nurse, Director at Lifestyle & Wellness, UK
Prof. Ayesha Minhas[15], Psychiatry, The Tree House, Rawalpindi, Pakistan
Prof.Farooq Naeem[16], Psychiatry, University of Toronto, Toronto, Canada
Prof. Haider Ali Naqvi[17], Psychistry, Dow University of Health Sciences, Karachi, Pakistan

For peer review only - http://bmjopen.bmj.com/site/about/guidelines.xhtml

Amna Noureen[2], Division of Child and Adolescents Mental health, Pakistan Institute of Living and Learning, Rawalpindi, Pakistan

Dr. Maria Panagioti[1],Division of Population Health, Health Services Research & Primary Care, Institute for Health Policy and Organisation /Alliance Manchester Business School, Manchester, UK

Prof.Anita Patel[18], Health Economics, Queen Mary, University of London, UK

Dr. Aaron Poppleton[1],NIHR Academic Clinical Fellow in General Practice, Center for Primary Care and Health Services Research,University of Manchester

Tinevimbo Shiri[19], Health Economics, Liverpool School of Tropical Medicine, England, UK

Dr. Mima Simic[20], Child and Adolescent Psychiatrist, The Maudsley Hospital London,NHS foundation England, United Kingdom

Dr. Sarwat Sultan[21], Division of Psychology, Bahauddin Zakaria University, Multan, Pakistan

Prof. Asad Tamizuddin Nizami[22], Psychiatry, Institute of Psychiatry, Rawalpindi, Pakistan

Prof. Zainab Zadeh[2] , Division of Child and Adolescent health, Pakistan Institute of Living and Learning, Karachi, Pakistan

Prof. Shehla Naeem Zafar[23], Community and Public Health Nursing, Iqra University, Karachi, Pakistan

Prof. Nasim Chaudhry[2] , Research and Development, Pakistan Institute of Living and Learning, Karachi, Pakistan

- Word count (excluding title page, abstract, references, figures, tables-4611/4000)
- Start & End Date of Study: March 2019 – Feburary 2023
- Protocol version V2-19th Nov'2021

For peer review only - http://bmjopen.bmj.com/site/about/guidelines.xhtml

ABSTRACT

Introduction: Suicide is a global health concern. Socio-cultural factors have an impact on self-harm and suicide rates. In Pakistan both self-harm and suicide are considered as criminal offense's and are condemned on both religious and social grounds. The proposed intervention "Youth Culturally Adapted Manual Assisted Problem Solving Training (YCMAP)" is based on principles of problem-solving and cognitive behavioural therapy (CBT). YCMAP is a brief, culturally relevant, scalable intervention that can be implemented in routine clinical practice if found to be effective.

Method and Analysis: A multi-centre rater blind randomized control trial (RCT) to evaluate the clinical and cost effectiveness of YCMAP including a sample of 652 participants, aged 12-18 years, presenting to general physicians/clinicians, emergency room after self harm or self referrals. We will test the effectiveness of 8 to 10 individual sessions of YCMAP delivered over three months compared to Treatment as Usual (TAU). Primary outcome measure is repetition of self-harm at 12 months. The seconday outcomes include reduction in suicidal ideation, hopelessness and disress and improvement in health related quality of life. Assessments will be completed at baseline, 3, 6, 9 and 12 month post randomization. The nested qualitative component will explore perceptions about management of self-harm and suicide prevention amongst adolescents and investigate participants' experiences with YCMAP. The study will be guided by the Theory of Change approach to ensure that the whole trial is centred around needs of the end beneficiaries as key stakeholders in the process.

Ethics and Dissemination: Ethics approval has been obtained from the Ethics Committee of University of Manchester, the National Bioethics Committee in Pakistan. The findings of this study will be disseminated through community workshops, social media, conference presentations and peer-reviewed journals.

Trial Registration Number: NCT04131179

For peer review only - http://bmjopen.bmj.com/site/about/guidelines.xhtml

STRENGTHS AND LIMITATIONS OF THIS STUDY

1. This is the first multi-centre RCT to evaluate clinical and cost effectiveness of a culturally relevant psychological intervention for young people presenting with self-harm in a Lower and Middle Income Country (LMIC).
2. Process evaluation will include in-depth interviews to understand the lived experiences of participants.
3. Stigma, fear of persecution by authorities, peer pressure and educational commitments may act as a barriers to young people participating in the study.
4. The Theory of Change approach will help mitigate some of the risks around stigma and refusal to participate by involving parents and young people from the inception of the trial.

INTRODUCTION

Suicide is one of the major public health concerns globally with 800,000 suicides each year across the globe (1). More than 75% of suicides occur in Low and Middle Income Countries (LMICs). However, there are now major concerns about increasing rates of self-harm and suicide among young people in High Income Countries (HIC) such as USA(2) and Europe (3). Overall, suicide is the 2nd leading cause of death in individuals between 15-29 years old (1). A recent review reported suicide rates in South Asia to be higher than the global average(4). However, such figures are likely to be an underestimate due to a lack of accurate data on suicide in Pakistan (5) and in many other LMICs (6).

In Pakistan, a conservative Islamic state, policies are changing but suicide and self-harm (defined below) remain criminal acts and are condemned both socially and religiously as a moral wrong. Suicide and self-harm are often considered taboo subjects across the country, thus contributing to a lack of evidence and under-reporting (4). There is, however, accumulating evidence that both self-harm and suicide rates have been increasing in Pakistan but there continues to be notable gaps in evidence(4). In a systematic review of mental health studies of adolescents in India, it was found that self-harm is particularly problematic in young people, with three months prevalence ranging from 3.9% to 25.4% in community based studies (7, 8). Self-harm is one of the strongest predictors of death by suicide (9). Self-harm also carries a substantial economic impact and has been associated with large treatment costs in Pakistan (10). Adolescents with a history of self-harm are at a higher risk of repeating this later in life (11)thus there is an important need to develop effective interventions in order to avoid long-term burden.

In adolescents, self-harm is the result of a highly complex interplay between genetic, biological, psychological, social, and cultural factors (11). Common set of risk factors and psycho-social mechanisms for self-harm within LMICs include being female, experiencing interpersonal

For peer review only - http://bmjopen.bmj.com/site/about/guidelines.xhtml

For peer review only

conflict, suffering from abuse (including domestic/family/gender based violence), hopelessness, and being diagnosed with psychiatric disorder (4). Other psychological influences include feelings of entrapment, a lack of belonging or connection (12). Research in Pakistan is imperative to explore the effectiveness of interventions for potential risk factors to reduce rates of self-harm and suicide (13). The World Health Organization (WHO) Report "preventing Suicide: A Global imperative" recommends "two-fold" public health approach for suicide prevention i.e. to identify the issues, and provide treatment for high-risk individuals.Considering the benefits of adult individual Cognitive Behaviour Therapy (CBT)-based psychotherapy(14), tested in our previous trial in Pakistan with positive results (15) and replicated in our recently completed trial with 901 adults participants in which we had to exclude 265 adolescents (less than 18 years) (CMAP2) (16). An unmet need was recognized to develop CMAP further for adolescent and adapting the similar brief psychological intervention for children and adolescents.Psychological therapies that work in western culture cannot always be implemented in a different culture and adaptation is considered to be essential (17, 18)

Since interpersonal conflicts with family members are a commonly reported precipitant of self-harm episodes in Pakistan (4, 13). It has been reported that problem-solving therapy "Culturally adapted Manual Assisted brief Psychological intervention (CMAP)" may be a useful intervention for prevention of self-harm in Pakistan (13). This psychological intervention, using variants of CBT that are age appropriate for the local culture and customs in Pakistan, may prove to be beneficial in reducing self-harm in adolescents (15). Before this intervention can be implemented at a larger scale, there is a need for establishing its clinical and cost-effectiveness via the proposed trial. We cannot assume that an intervention developed for adults would be suitable for children and adolescents and since self-harm remains a major problem in young people, this planned trial fills an important gap and clinical need.

METHODS AND ANALYSIS

Objectives

This study includes both quantitative and qualitative aspects.

The objectives of the quantitative component are:

1. To determine the clinical effectiveness of YCMAP over 12 months based on the following outcomes:
    a. Primary outcome:
        i. To measure the effectiveness of YCMAP in comparison to treatment as usual (TAU) in terms of repetition of self-harm 12-month post

For peer review only - http://bmjopen.bmj.com/site/about/guidelines.xhtml

randomization. This will be assessed using adapted Suicide Attempt Self-Injury Interview SASII (19).

b. Secondary outcomes:
    i. Suicidal ideation will be assessed using the "Beck Scale for Suicidal ideation (BSI)"(20).
    ii. Feelings of hopelessness will be assessed using the "Beck Hopelessness Scale (BHS)"(21).
    iii. Level of distress will be assessed using a 10-item scale the "Kessler Psychological Distress Scale (K10)"(22).
    iv. EQ-5D- Y will be used to assess "health related quality of Life" (23).
    v. Client Satisfaction Questionnaire-"CSQ-8" will be used to assess participants' satisfaction with services (24).

Translated versions of all these scales have been developed and used in previous trials CMAP1 (15) and CMAP 2 (13) by following established protocols for translating such measures (25).The scales which were not available in Urdu were translated for an MPhil project (Brief psychological intervention for adolescents who self harm, IPP/BU/ER/103/1377) and reviewed in initial Patient and Public Involvement and Engagement meetings.

Assessments will be carried out at baseline, completion of the intervention (3 months), 6, 9 and 12 months after randomisation. See figure 1 below

2. To determine the cost effectiveness of the YCMAP intervention over 12 months.

The objectives of the qualitative component are:

1. To explore the experience of participants with YCMAP and their perception about benefits and negative or adverse consequence of it.
2. To explore in detail participants' reasons for continuing (completer) or not continuing (drop out) with the study.
3. To explore clinicians and other stakeholder views (such as school teachers) on the YCMAP and its impact.
4. To explore therapists' perspective on the delivery of the YCMAP.

The expected outputs of the study are:
a) A manual-assisted evidence based intervention - the YCMAP, ready for integration into the health care services.

For peer review only - http://bmjopen.bmj.com/site/about/guidelines.xhtml

b) Standardized training and supervision package - a resource pack and training program on 'how to do it' for use across health services.

c) Information Tool-kit for the families and the wider community. This will be service-user defined and will be used to increase awareness about mental health in general and self-harm and suicide in young people in particular.

Inclusion Criteria

Adolescets meeting the study inclusion criteria will be invited to take part in the study by their primary care clinician, ward or emergency room (ER) clinician who is making the initial assessment. In the context of this study, self-harm is defined as: *"an act with non-fatal outcome, in which an individual deliberately initiates a non-habitual behaviour that, without interventions from others, will cause self-harm, or deliberately ingests a substance in excess of the prescribed or generally recognised therapeutic dosage, and which is aimed at realizing changes which the subject desired via the actual or expected physical consequences"(26).*

- Age: 12-18 years.
- History of recent self-harm which is defined as " self-harm occurring within the last 3 months (from the initial identification of a potential participant)". This period is considered as high risk for repetition in young people (11).
- Resident in the trial catchment area.
- All participants known to clinical or health services. This will be necessary to ensure all participants have access to TAU.

Exclusion Criteria

- Patients with a severe mental illness such as schizophrenia spectrum and other psychotic disorders, bipolar and severe depressive disorder will be excluded. This intervention has been developed to help individuals who experience common mental health problems such as anxiety or mild to moderate depressive disorders. It has not been developed to work with people with severe mental health difficulties such as psychotic disorders. We consider that the intervention would require work with service users and carers and further adaptation work with people who have severe mental health illness.
- Patients with conditions limiting engagement with assessment/intervention.
- Temporary resident unlikely to be available for follow up.

Design

A multi-centre rater blind RCT with randomisation by individual participants in order to compare the YCMAP in addition to TAU with TAU alone.

For peer review only - http://bmjopen.bmj.com/site/about/guidelines.xhtml

Study Site and Population

Study sites are all participating primary care clinics,emergency departments and medical wards of general hospitals in five major cities of Pakistan, Karachi, Hyderabad, Lahore, Multan and Rawalpindi.

Sample Size

The sample size is based on the primary outcome, repetition of self-harm in a 12-month period (yes/no). The TAU arm of the study has an expected self-harm rate of 20% (7). A clinically important effect would be a reduction to 7.5% in the intervention group. Under these presumptions, and assuming a 5% significance level and 90% power, a study with no clustering would require 158 patients per arm. The study has a partially nested design due to therapist clustering in the YCMAP arm. Our sample size calculation has taken account of this by adjusting the sample size upwards, conservatively assuming clustering in both arms. Based on previous analysis of therapist trials, we believe that the intraclass correlation coefficient (ICC) is likely to have a value between 0.01 and 0.05 for this type of outcome measure. Assuming an ICC of 0.05, and a cluster size of 16 patients per therapist, a design effect of 1.75 is calculated. This increases the numbers required to 277 per arm. Furthermore,based on our previous work there is expected to be a 15% loss to follow-up, and so the final numbers recruited will be 326 per arm, with a total sample of 652 participants.

Randomisation

The researcher will complete a checklist, confirming eligibility, obtain written (or verbal audio-recorded consent from the subject due to current pandemic COVID-19 SOPs), and complete the baseline measures. The researcher will contact the randomisation centre, who will re-check eligibility, record baseline measures and assign a participant's trial number. Treatment assignment will then be determined using stochastic minimisation controlling for gender, age and type of self-harm behaviour. The independent statistician would inform the project manager of the random assignments, but this information would not be shared with research staff, so that they remain unaware of whether a particular participant receives YCMAP or not. Participants assigned to the YCMAP would then be contacted by a therapist, who will arrange an initial meeting within 2-4 weeks after baseline to start the intervention. Participants in the TAU group would be informed of their allocation to this group after randomisation had taken place.

Recruitment and Baseline Assessment

For peer review only - http://bmjopen.bmj.com/site/about/guidelines.xhtml

Potentially eligible individuals will be identified by clinical staff at the participating recruitment sites. An age appropriate version of the Participant Information Sheet (PIS) will be provided alongside the standard PIS, so both the parent/guardian and child can access this information. For individuals who cannot read, information about the study will be summarised by the clinician for them. The consent was obtained from the parent/guardian along with consent from the participating young person (The Nuffield Council for Bioethics guidance suggests that consent rather than assent should be sought from young people in research).Therefore, it is ensured and clearly mentioned in the participant information sheet for both the young people and their parents that it is mandatory for parents/guardians to consent for young person to participate in the study.

Intervention

YCMAP is a "Youth culturally adapted manual assisted psychological intervention" based on CBT principles. It comprises of 8-10 sessions delivered over three months.The first eight sessions are offered weekly and further sessions fortnightly on a one to one basis and each session lasts for about 60 minutes. The YCMAP has been culturally adapted with permission from "CMAP" (15), "Life after self-harm" (27) and "Cutting down: A CBT workbook for treating young people who self-harm"(28). The intervention includes psycho-education and a comprehensive cognitive behavioural assessment of the self-harm attempt using virtual stories of four young people. The therapy focuses on current problems that contributed to the self-harm episode. Therapists and adolescent clients choose from a list of techniques those which are most relevant to the client's problems. Therapy is therefore adapted to fit with the clients problems and primarily utilises problem solving, CBT, and dialectical therapy strategies to bring about change.

To help determine the most appropriate coping strategy a coping tree is designed. Training in assertiveness and anger management are offered to help the young person to develop resilience to cope with stress. To the existing YCMAP we will also aim to review the look and feel of the resource by including licenced content from My Big Life - a course developed by our collaborative partner Five Areas Limited. This accessible, story-based approach uses illustrations of young people facing different scenarios at home and at school to illustrate key CBT-based concepts: Understanding your feelings -How to get a Big Life (behavioural activation) - Thinking in a Big Life way (identifying and changing thoughts that upset and affect how you feel) - Relaxation approaches - Building inner confidence - Practice scenarios - Trainer notes and linked worksheets/prompt cards and posters. Asian versions of the course already exist with amended artwork. As part of the development programme selected content will be added to the course and trainer notes will be modified (based on feedback) as needed.

All research staff will be required to have at least an undergraduate level degree in a relevant area (e.g. psychology) and will be trained in "Good Clinical Practice (GCP) " in research, consent process, and use of the assessment measures. Regular supervision meetings will be arranged for

For peer review only - http://bmjopen.bmj.com/site/about/guidelines.xhtml

research staff and therapists for case discussions, identifying and managing distress in participants. A study protocol will be in place prior to the start of the trial. This will include details about how to manage difficult situations arising in research, safety and lone working arrangements.

Treatment as Usual (TAU)

Local psychiatric, medical and primary care services provide standard routine care according to their clinical judgment and available resources. We will administer Client Services Receipt Interview (CSRI) (29) to obtain details regarding treatment received by each study participant.These participants will receive an initial assessment along with TAU as ascertained by their treating doctor at the hospital or their GP. The current practice is that self-harm patients are not routinely referred to mental health services. Research staff will record the nature and intensity of the routine care delivered for each participant.

Patient and Public Involvement and Engagement (PPIE) Group

This group will help to ensure that the research agenda for self-harm and suicide prevention studies is informed by and aligned with service users and carer priorities.

Statistical Analysis

Statistical analysis will be based on intention-to-treat principles, subject to the availability of data. During the course of the trial, periodic random quality checks of data will be carried out by the trial statistician blind to treatment allocation. Once data entry has been completed, preliminary data analysis will be carried out blind to treatment allocation, prior to un-blinding. The results of the trial will follow the standard CONSORT recommendations. Baseline and follow-up data will be summarised using the appropriate descriptive statistics and graphical summaries. Treatment effects will be presented with 95% confidence intervals. We will investigate baseline factors that predict nonresponse using a logistic random effects model as non-response may be clustered by therapy group. The statistical analysis of the primary outcome measure (repetition of the self-harm episode within 12-months) will be based on a logistic random effects model with randomised treatment, age, gender and type of self-harm as fixed effects and a random effect of therapist in the treatment arm, with individuals in the control arm being treated as clusters of size 1. The continuous secondary outcome measures will calculate treatment effects using a linear mixed model, with a random effect of therapist and the set of baseline covariates as above including baseline values of the outcome where available. A single model will be fitted across all time-points, with a fixed effect for time, and interactions between time and treatment group. There will

For peer review only - http://bmjopen.bmj.com/site/about/guidelines.xhtml

be no adjustment to secondary outcomes CIs for multiple testing. Binary secondary outcomes will take a similar approach to continuous outcomes, but will use logistic random effects models.

Economic Evaluation

The economic evaluation will take a societal perspective that incorporates the costs of both formal and informal health care and other relevant economic impacts such as on education. It will include financial impacts on providers and out of pocket payments by participants and their families. The use and payment for services will be collected using the CSRI (29) with patients and their families at baseline and at each follow up assessment. The use of the YCMAP intervention will be recorded separately by the trial team. Appropriate unit costs will be collected and attached to individual-level resource use quanitities to estimate total care costs for each individual. Cost estimates for the YCMAP intervention will include a) the salary of the therapists and b) indirect costs like session preparation, supervision, on-costs and capital overheads. Comparisons of total costs between the YCMAP and TAU arms will be based on non-parametric bootstrapped regressions (with co-variates for baseline costs, outcome measures and other key baseline factors) to account for the likely non-normal distribution in costs. An inital analysis will examine whether any additional costs associated with the YCMAP intervention are offset by savings elsewhere.

More formal assessments of cost-effectiveness will link between-arm differences in average costs with between-arm differences in a) the primary outcome measure, repetition of self-harm (SASII) and b) quality adjusted life year (QALY) gains. QALYs will be estimated by applying relevant utility weights to health states measured through EQ-5D-Y (23, 30). Costs and outcomes will be linked in the form of incremental cost-effectiveness ratios (ICERs) where relevant, and cost-effectiveness acceptability curves (CEACs) based on the net benefit approach. As per the clinical effectiveness analyses, we will use an intention-to-treat approach for all economic analyses.

Qualitative Data Analysis

A purposefully selected subset (stratified by age, gender and self-harm severity) of participants in the treatment group will be invited to complete a qualitative one-to-one digitally recorded interview (face to face or via phonecall) lasting 1-1.5 hours. This will focus on their experience with the YCMAP intervention, the barriers and facilitators to engagement and perceived positive or negative experience with the intervention. A sample of 12-20 participants is likely to be sufficient to ensure data saturation (31) (32). Focus groups ($k = 2$ with 8-10 participants each) with therapists and key stakeholders will also be conducted to enable a further process analysis concerning the wider implementation of YCMAP into the Pakistani health systems. During both the individual and group interviews, broader suggestions for management and prevention of self-harm among young Pakistanis will also be collected to inform future service and research development.

For peer review only - http://bmjopen.bmj.com/site/about/guidelines.xhtml

We will explore perspectives of therapists and their supervisors about training and delivery of the model of care. We will also interview other key stakeholders to establish facilitators and barriers to implementation of the intervention in Pakistan.Targeted interviews will be done with a sample of participants who completed the intervention and a sample of participants who 'dropped out' before completion. Up to 30 interviews are likely to be needed with patient participants, to achieve category saturation. New text mining tools will be used to link heterogeneous data, to ensure that knowledge scattered with in lengthly textual data reaches clinicians, patients and policy makers to support decision making using different sources of information (33). Qualitative data will be analysed using Thematic Analysis (34) adopting a critical realist perspective (35).This will enable the research team to draw conclusions on the intervention whilst accounting for contextual and cultural factors.

Topic guides will be developed through discussion amongst trained qualitative researchers as well as with reference to relevant literature. Semi- structured interviews will be done to explore views on the "effectiveness and sustainability" of the YCMAP intervention in the management of people presenting with self-harm. Written or verbal consent at the time of interview (face to face or telephone) or prior to the interview (considering current pandemic) will be obtained from study participants and stake holders.

Internal pilot study

We have included an internal pilot phase (initial 12 months) with clear stop/go criteria across the study centres. This is included to ensure achievement of recruitment targets is viable across all sites included in this full trial. Progression to the full trial will be dependent on meeting the key objectives and stop/go criteria outlined below. The pilot phase will examine the processes for recruitment and logistical practices to support the effective execution of the full RCT. The internal pilot phase will be conducted in line with the National Institute of Health Research (NIHR) guidelines (36). The key objectives of the pilot are as follows; i) Recruitment of sufficient number of General Practitioners/Hospital Departments to support the trial. ii) Identify the proportion of eligible participants recruited across the study centres, and examine the feasibility of achieving recruitment targets to a full multicenter RCT. The pilot will be judged successful if sufficient GPs and hospitals are recruited to enroll 200 participants in the first year of recruitment i.e 4-12 months.We will monitor recruitment and identify the reasons for any shortfall on a monthly basis. The predetermined Stop-Go criteria is as follows:

i. Go if successfully recruit 180 (target minus 10%) or more participants during pilot study phase

ii. Rescue plan to be implemented if less than 180 but greater than 120 participants recruited in pilot phase.

For peer review only - http://bmjopen.bmj.com/site/about/guidelines.xhtml

iii. Stop if less than 120 participants recruited into the trial.

Theory of Change (ToC)

The study team uses ToC as the standard framework for all research projects. The overall study will be underpinned by the ToC causal model of planning, monitoring, evaluation and impact assessment to ensure that marginalised voices are included in developing the vision, identifying barriers and challenges from the lens of the end beneficiaries and short, medium and long term outcomes are developed and delivered so real change happens from the perspective of the target group. A ToC workshop with key stakeholders has already taken place to ensure all stakeholders have a buy in and ownership of the process. See Figure 2

The ToC causal diagram as an output is evidence of stakeholder engagement and ownership. Also,ToC will enable us to show how trial results are adopted in wider practice and on what basis,what barriers and challenges were faced during the trial and what assumptions were made in defining the goal statements? Informed by the ToC workshop, a Young People's Advisory Group will be set up to provide insight into the lived experiences of today's youth in Pakistan.

As religiosity was a dominant thread from the community engagement exercise, there was recognition that spiritual advice was often the first point of contact for patients rather than health professionals. The trial was not designed to try to replace such religious norms but to work alongside them while recognising that there could be delays in referrals. There was also no restriction on accessing spiritual guidance making professional psychological therapies an addition not a substitute for such support. Importantly, mental health concerns are stigmatising and suicide is still considered as a criminal offense.

Working with religious leaders was therefore a critical part of community engagement with Y-CMAP and its evaluation through the RCT the concept of 'dawa and dua' medicine and prayer going hand in hand as part of therapy tend to promote adherence and engagement.

COVID-19 Continuity Plan

Due to global pandemic COVID-19, amendments to study procedures in response to the lock down and social distancing in Pakistan were made to ensure smooth running of the project. See supplementary file.

Trial Steering Committee (TSC)

We will convene an independently chaired TSC to approve and provide oversight of the trial throughout its various stages. The TSC will include the chair, user representative, three PIs, an

For peer review only - http://bmjopen.bmj.com/site/about/guidelines.xhtml

independent statistician and a representative from the local health department. This will be independent of the trial management team. The (TSC) will convene annually, but twice in the first year, with feedback from the Chair as and when needed.

Data Monitoring and Ethics Committee (DMEC)

The DMEC will oversee the data and advise the TSC on any ethical or safety concerns.

Ethics and Dissemination

Ethics approval for the trial has been obtained from the Research Ethics Committee of University of Manchester (Ref: 2019-5024-10755) and the National Bioethics Committee in Pakistan (Ref: No.4-87/NBC-419/19/1213). The study findings will be disseminated using social media, at national and international conferences in partnership with the “service user”, “carer” and “community organizations”. Also, study results will be submitted to peer and non peer-reviewed journals for publication.

Discussion

There has been a recent significant increase in number of trials related to self-harm and suicide prevention which reflects the need and international concern about self-harm/suicide prevention. However, in Pakistan evidence based research is limited with no specific intervention for adolescents with self harm history. The evidence suggests that CMAP intervention for adults is effective in Pakistan (15). The components of CMAP intervention may also be beneficial for adolescents presenting after self-harm, therefore there is a strong need to determine the effectiveness of culturally adapted manual assisted problem solving intervention for adolescents in Pakistan. Findings will also contribute to evidence based treatments for self-harm and will meet important clinical needs of Pakistan and will help guide policy makers in developing suicide prevention policies.

Authors Contributions

NH, NC, TK, IBC and ST involved in conceptualisation, designing and planning of the project (including protocol development). EC, SA reviewed qualitative component. ZZ, CW, PT, KD and FN reviewed intervention section, RM facilitated the ToC process. MA, RE reviewed statistical analysis plan and AP, AG reviewed economic evaluation plan. All authors SA, SoA, MHA, AB, SuA, SE, JG, KH, FJ, AK, TM, AnMc, AM, HAN, AN, MP, AaP, TS, MS, SS, ATN, and SNZ gave their valuable inputs in finalizing the study protocol.

Funding Statement

For peer review only - http://bmjopen.bmj.com/site/about/guidelines.xhtml

The Medical research Council /DFID/NIHR programme (MR/R022461/1) has funded this trial. The funder has no role in designing the study methodology, data analysis and interpretation, manuscript writing and in the decision to submit the protocol for publication.

Competing Interests

All authors have read and declared that we have no competing interests with the exception of NH, NC, IBC, TK and CW. NH, former Trustee of "Pakistan Institute of Living and Learning (PILL)", "Abaseen Foundation (UK)" and "Lancashire Mind (UK)". At "Manchester Global Foundation", he is the Chair of Board of Trustees. He is also a member of the executive committee for the Faculty of Academic Psychiatry, at the Royal College of Psychiatrists. NH is a NIHR Senior Investigator. NC is Associate Director of Global Mental Health and Cultural Psychiatry Research Group. IBC, former Trustee of "PILL" is Honorary Professor at the University of Manchester. NH, IBC and NC have received support for educational programs and/or travel support and/or speaker fees from pharmaceutical companies. CW is the author of a book aimed at suicide prevention, and has written a range of books and online CBT-based course resources that are available as both, free access and on a commercial basis. NH, NC, IBC and TK's time is partially funded by the Global Challenges Research Fund "South Asia Harm Reduction Movement-SAHAR M" (MR/P028144/1).

Acknowledgement

We express our gratitude to all team members, researchers, consultants and investigators for their support and advice in the development and setting up of the trial. We would like to thank community gate keepers, service uers and patient and public involvement group members for their kind support, input and feedback on the development of the funding application, spreading awareness among masses and in trial recruitments.

We would also like to thank Trial Steering Committee (TSC) members, Prof. David Kingdon (Chair), Dr. Mehmood Shaukat, Faraz Usman and Data Management and Ethics Committee (DMEC) members Prof. Unaiza Niaz (Chair), Dr. Qamar Saeed and Dr. Maryum Ilyas for overseeing the trial's progress and management.

For peer review only - http://bmjopen.bmj.com/site/about/guidelines.xhtml

References:

1. WorldHealthOrganization. Preventing suicide: A global imperative: World Health Organization; 2014.
2. Twenge JM, Joiner TE, Rogers ML, Martin GN. Increases in depressive symptoms, suicide-related outcomes, and suicide rates among US adolescents after 2010 and links to increased new media screen time. Clinical Psychological Science. 2018;6(1):3-17.
3. Castelpietra G, Knudsen AKS, Agardh EE, Armocida B, Beghi M, Iburg KM, et al. The burden of mental disorders, substance use disorders and self-harm among young people in Europe, 1990–2019: findings from the Global Burden of Disease Study 2019. The Lancet Regional Health-Europe. 2022;16:100341.
4. Shekhani SS, Perveen S, Akbar K, Bachani S, Khan MM. Suicide and deliberate self-harm in Pakistan: a scoping review. BMC psychiatry. 2018;18(1):1-15.
5. Farooq U, Majeed M, Bhatti JA, Khan JS, Razzak JA, Khan MM. Differences in reporting of violence and deliberate self harm related injuries to health and police authorities, Rawalpindi, Pakistan. PloS one. 2010;5(2).
6. Jordans MJ, Kaufman A, Brenman NF, Adhikari RP, Luitel NP, Tol WA, et al. Suicide in South Asia: a scoping review. BMC psychiatry. 2014;14(1):358.
7. Aggarwal S, Berk M. Evolution of adolescent mental health in a rapidly changing socioeconomic environment: A review of mental health studies in adolescents in India over last 10 years. Asian journal of psychiatry
2015;13:3-12.
8. Aggarwal S. Suicide in India. J British medical bulletin. 2015;114(1).
9. Ribeiro J, Franklin J, Fox KR, Bentley K, Kleiman EM, Chang B, et al. Self-injurious thoughts and behaviors as risk factors for future suicide ideation, attempts, and death: a meta-analysis of longitudinal studies. Psychological medicine. 2016;46(2):225-36.
10. Shahid M, Khan MM, Naqvi H, Razzak J. Cost of treatment of deliberate self-harm: a study from Pakistan. Crisis. 2008;29(4):213-5.
11. Hawton K, Saunders KE, O'Connor RC. Self-harm and suicide in adolescents. The Lancet Neurology. 2012;379(9834):2373-82.
12. Klonsky ED, May AM. The three-step theory (3ST): A new theory of suicide rooted in the “ideation-to-action” framework. International Journal of Cognitive Therapy. 2015;8(2):114-29.
13. Husain MO, Umer M, Taylor P, Chaudhry N, Kiran T, Ansari S, et al. Demographic and psychosocial characteristics of self-harm: The Pakistan perspective. Psychiatry research. 2019;279:201-6.
14. Witt KG, Hawton K, Hetrick S, Taylor Salisbury T, Townsend E, Hazell P. Interventions for self-harm in children and adolescents. Cochrane database of systematic reviews. 2020.
15. Husain N, Afsar S, Ara J, Fayyaz H, ur Rahman R, Tomenson B, et al. Brief psychological intervention after self-harm: randomised controlled trial from Pakistan. The British Journal of Psychiatry. 2014;204(6):462-70.

For peer review only - http://bmjopen.bmj.com/site/about/guidelines.xhtml

16. Kiran T, Chaudhry N, Bee P, Tofique S, Farooque S, Qureshi A, et al. Clinicians' Perspectives on Self-Harm in Pakistan: A Qualitative Study. Frontiers in psychiatry. 2021;12:484.
17. Degnan A, Baker S, Edge D, Nottidge W, Noke M, Press C, et al. The nature and efficacy of culturally-adapted psychosocial interventions for schizophrenia: a systematic review and meta-analysis. Psychological Medicine. 2018;48(5):714-27.
18. Rathod S, Gega L, Degnan A, Pikard J, Khan T, Husain N, et al. The current status of culturally adapted mental health interventions: a practice-focused review of meta-analyses. Neuropsychiatric disease treatment. 2018;14:165.
19. Linehan MM, Comtois KA, Brown MZ, Heard HL, Wagner A. Suicide Attempt Self-Injury Interview (SASII): development, reliability, and validity of a scale to assess suicide attempts and intentional self-injury. Psychological assessment. 2006;18(3):303.
20. Beck AT, Steer RA. Manual for the Beck scale for suicide ideation. San Antonio, TX: Psychological Corporation
1991;63.
21. Beck AT, Steer RA, Pompili M. BHS, Beck hopelessness scale: manual: Psychological corporation San Antonio, TX; 1988.
22. Andrews G, Slade T. Interpreting scores on the Kessler psychological distress scale (K10). Australian and New Zealand journal of public health. 2001;25(6):494-7.
23. Ravens-Sieberer U, Wille N, Badia X, Bonsel G, Burström K, Cavrini G, et al. Feasibility, reliability, and validity of the EQ-5D-Y: results from a multinational study. Quality of life research. 2010;19(6):887-97.
24. Attkisson CC, Zwick R. The Client Satisfaction Questionnaire: Psychometric properties and correlations with service utilization and psychotherapy outcome. Evaluation program planning. 1982;5(3):233-7.
25. Rahman A, Iqbal Z, Waheed W, Husain N. Translation and cultural adaptation of health questionnaires. Pakistan Medical Association. 2003;53(4):142-6.
26. Platt S, Bille-Brahe U, Kerkhof A, Schmidtke A, Bjerke T, Crepet P, et al. Parasiticide in Europe: the WHO/EURO multicentre study on parasuicide. I. Introduction and preliminary analysis for 1989. Acta Psychiatrica Scandinavica. 1992;85(2):97-104.
27. Schmidt U, Davidson K. Life after self-harm: A guide to the future: Routledge; 2004.
28. Schmidt U, Taylor L, Simic M. Cutting Down: A CBT workbook for treating young people who self-harm: Routledge; 2015.
29. Beecham J, Knapp M. Costing psychiatric interventions. Measuring mental health needs. 2001;2:200-24.
30. Brooks R, Group E. EuroQol: the current state of play. Health Policy & Planning. 1996;37(1):53-72.
31. Guest G, Bunce A, Johnson L. How many interviews are enough? An experiment with data saturation and variability. Field methods. 2006;18(1):59-82.
32. Kuzel AJ. Sampling in qualitative inquiry. 1992.
33. Zerva C, Nghiem M-Q, Nguyen NT, Ananiadou S. Cited text span identification for scientific summarisation using pre-trained encoders. Scientometrics. 2020;125(3):3109-37.
34. Braun V, Clarke V, Terry G. Thematic analysis. Qual Res Clin Health Psychol: 95–114. 2014.
35. Fletcher AJ. Applying critical realism in qualitative research: methodology meets method. International Journal of Social Reserach Methodology. 2017;20(2):181-94.
36. Hampson L, Williamson P, Wilby M, Jaki T. Recruitment progression rules for internal pilot studies monitoring recruitment. Trials. 2015;16(2):1-.

For peer review only

For peer review only - http://bmjopen.bmj.com/site/about/guidelines.xhtml

Figure:1
Flow Diagram of the YCMAP study

For peer review only

For peer review only - http://bmjopen.bmj.com/site/about/guidelines.xhtml

For peer review only

Figure: 2
Theory of Change

For peer review only - http://bmjopen.bmj.com/site/about/guidelines.xhtml

Supplementary File
COVID 19 Continuity plan

For peer review only

For peer review only - http://bmjopen.bmj.com/site/about/guidelines.xhtml

Figure 1
Flow diagram of the YCMAP study

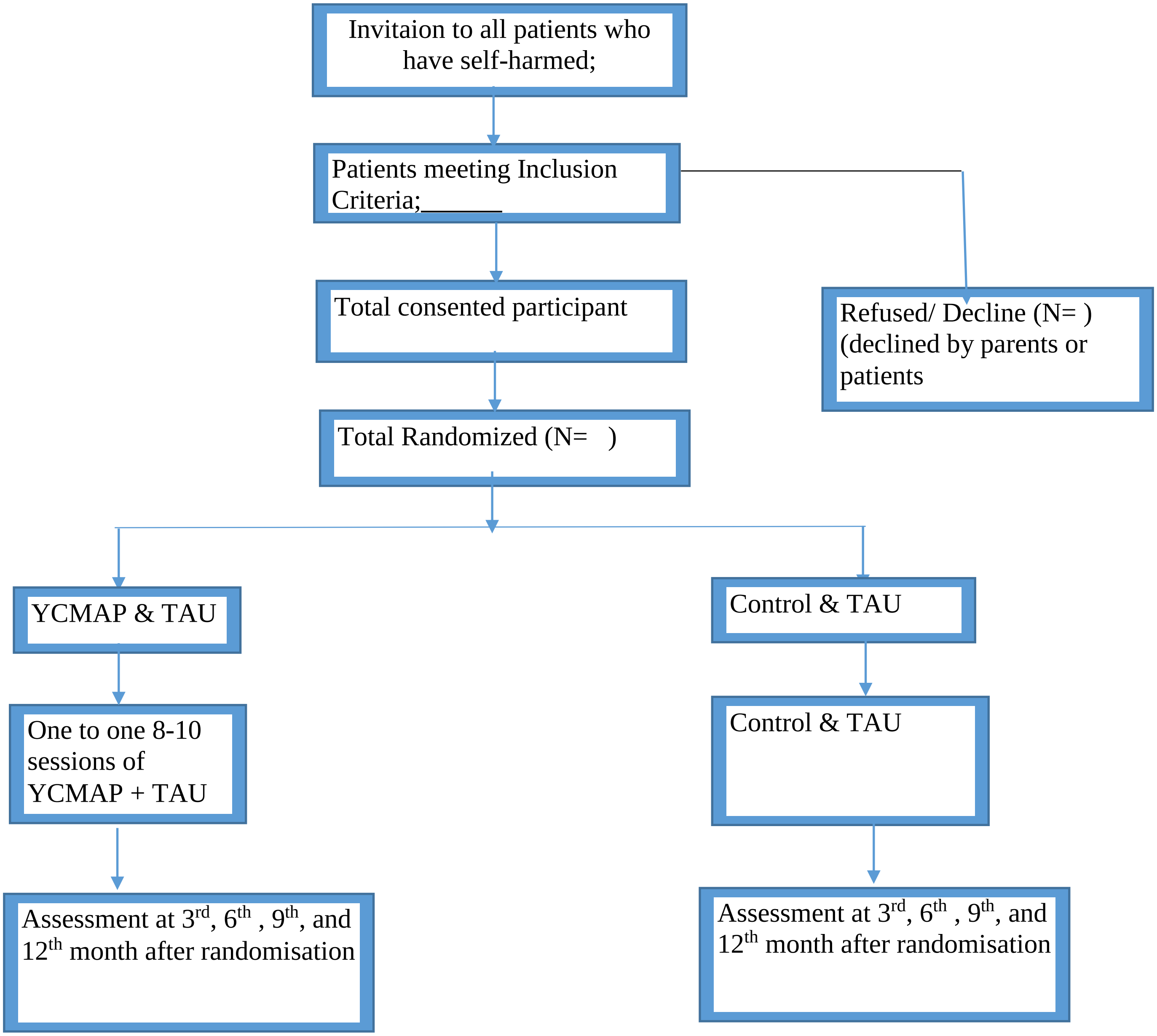

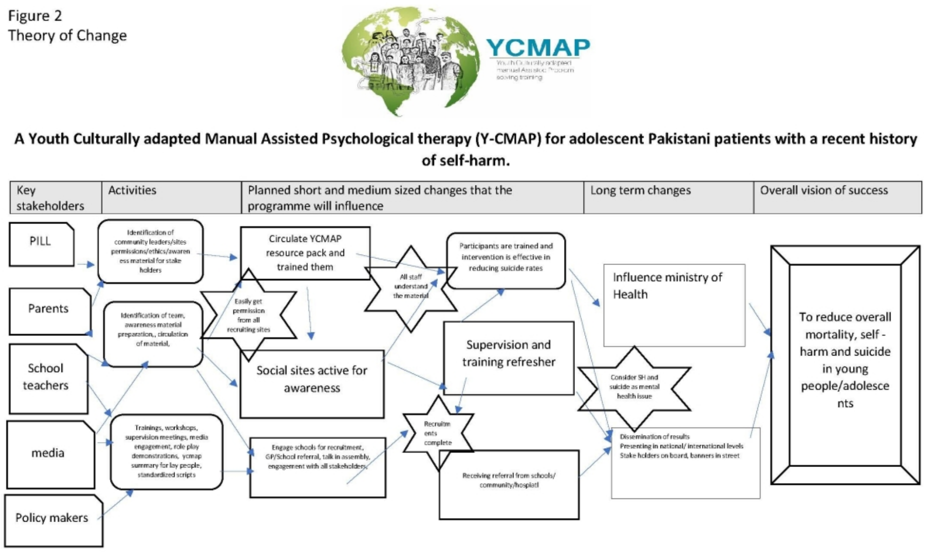

Theory of Change

297x210mm (200 x 200 DPI)

COVID-19 continuity plan

In March 2020, due to the first wave of global pandemic-COVID-19 there was a need to make amendments to study procedures in response to the lock down and social distancing in Pakistan. The trial during this period was in the internal pilot phase and in order to ensure smooth running of the project certain changes were made to continue with collection of data, follow ups and delivery of the intervention.

Follows ups (3rd, 6th, 9th and 12th month after randomization):

Initial ethical approval was obtained for face to face assessment and intervention. However, after COVID-19, we submitted ethics amendment for data collection and delivery of intervention digitally (i.e. through telephone call, zoom, skype or other remote means) so that the study team could continue their work and meet targets on time with working from home condition. The consent form that was previously completed on paper further consents will be obtained remotely and the assessment measures to be completed remotely as well. Recordings of consent will be stored in password protected systems.

YCMAP Intervention:

YCMAP intervention comprises of 8-10 sessions delivered in a face to face setting, usually in research offices, community clinics and homes (as per participant choice). As of 20th march 2020, due to lock down across Pakistan it was difficult to deliver face to face sessions. To comply with preventive measures such as social distancing and lock down guidelines, the YCMAP intervention will be delivered remotely, via a phone call, WhatsApp call, zoom or skype rather than in person face to face sessions. Contingency plan will be implemented to manage confidentiality and safe guarding risks.

Qualitative Interviews:

Qualitative interviews will be completed face to face or through telephone. Due to the pandemic situation COVID-19, topic guides will be revised in order to explore the experience of change in mode of delivery of intervention as well as impact of Covid 19 after discussing it with supervisors (EC, NC).

Supervision**:**

Arrangements for supervision of the therapists will continue as per protocol. The supervisions will continue to be carried out remotely via video link.

For peer review only

Trainings:

Arrangements for training of YCMAP team members will continue and carried out remotely via video link

For peer review only
